# Synthesis, antifungal and antibacterial activity for novel amide derivatives containing a triazole moiety

**DOI:** 10.1186/1752-153X-7-30

**Published:** 2013-02-12

**Authors:** Ruping Tang, Linhong Jin, Chengli Mou, Juan Yin, Song Bai, Deyu Hu, Jian Wu, Song Yang, Baoan Song

**Affiliations:** 1 State Key Laboratory Breeding Base of Green Pesticide and Agricultural Bioengineering, Key Laboratory of Green Pesticide and Agricultural Bioengineering, Ministry of Education, Guizhou University, Guiyang, China; 2 Research and Development Center for Fine Chemicals, Guizhou University, Guiyang, 550025, China

## Abstract

**Background:**

Plant fungi (e.g., *Pellicularia sasakii, Gibberella zeae, Fusarium oxysporum,* and *Cytospora mandshurica* and *Phytophthora infestans*) and bacteria (e.g., *Ralstonia solanacearum*) are extremely difficult to manage in agricultural production. The high incidence of plant mortality and the lack of effective control methods make *P. sasakii* and *R. solanacearum* two of the world’s most destructive plant pathogens. Pathogenic fungi and bacteria are responsible for billions of dollars in economic losses worldwide each year. Thus, we designed an active amide structure and synthesized a series of novel amide derivatives containing a triazole moiety to discover new bioactive molecules and pesticides that can act against fungi and bacteria.

**Results:**

A series of amide derivatives containing a triazole moiety were synthesized. All the obtained compounds were characterized through proton and carbon nuclear magnetic resonance spectroscopy, infrared spectroscopy, and elemental analysis. Preliminary antifungal activity test showed that some of the synthesized compounds exhibited moderate antifungal activity against *P. sasakii*, *G. azeae*, *F. oxysporum*, *C. mandshurica*, and *P. infestans* at 50 mg/L. Compound **4u** displayed more potent antifungal activity against *P.* sasakii and *G. azeae* than hymexazol. Preliminary antibacterial activity results showed that some of the synthesized compounds exhibited high anti-bacterial activity against *R. solanacearum* at 200 mg/L. Compounds **4m** and **4q** displayed high antibacterial activity against *R. solanacearum*, with 71% and 65% inhibitory rates, respectively.

**Conclusions:**

A series of novel amide derivatives containing 1,2,4-triazole moiety were synthesized through the reaction of intermediate **3** with different acyl chlorides and anhydrous potassium carbonates in anhydrous tetrahydrofuran at 50°C, using 2,4-dichloroacetophenoneas as a starting material. The title compounds exhibited high inhibitory effects against *P. sasakii*, *R. solanacearum*, and *G. azeae*.

## Background

**Figure 1:**
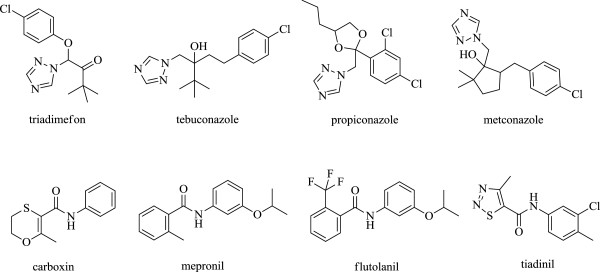
Commercialized fungicides containing 1,2,4-triazole or amide substructures.

1,2,4-Triazole, an important class of heterocyclic rings, has attracted increasing attention due to their broad activities, such as fungicidal [1,2], insecticidal [3,4], herbicidal [5,6], and bactericidal [7,8]. It may also serve as a plant growth regulatory agent [9] and has excellent potential in the pesticide field. Since the discovery of triadimefon by Bayer in 1976, 1,2,4-triazole has been used as fungicide for approximately 30 years. It quickly gained a significant importance in the protection of various crops, representing a significant progress in the chemical control of fungal diseases. With the increasing number of triazole derivatives, several compound containing tebuconazole, propiconazole, and metconazole have been developed and commercialized, respectively (Figure 1).

Since the first synthesis of carboxin by Schmeling and Kulkain in 1966 [10], amide fungicides have also been used for controlling plant diseases for more than 40 years. Amide derivatives have become a research hot spot in the development of pesticides because of their high-efficiency active features and broad spectrum bioactivities, such as antifungal [11,12], insecticidal [13], and herbicidal [14]. Currently, some amide derivatives have been developed and commercialized as pesticides. Mepronil, flutolanil, and tiadinil are known for their ability to protect certain plants from severe diseases and pests (Figure 1). In our recent publications [15,16], several pyrazole amide derivatives containing a hydrazone moiety have been synthesized and tested for their antifungal activity. The synthesized compounds exhibited antifungal activity against *Fusarium oxysporum* and *Cytospora mandshurica*, with inhibitory rates ranging from 40.82% to 50.32%. In addition, some hydrazone derivatives containing a pyridine moiety possessed high antibacterial activity against *Ralstonia Solanacearum*[16].1-(2,4-Dichlorophenyl)-3-aryl-2-(1*H*-1,2,4-triazol-1-yl) prop-2-en-1-one derivatives have been synthesized using aldol condensation between 1-(2,4-dichlorophenyl)-2-(1*H*-1,2,4-triazol-1-yl) ethan one and an aryl aldehyde. The better compound showed an antifungal activity level similar to that displayed by hymexazol against *Gibberell azeae*, *F. oxysporum*, and *C. mandshurica*[17].

The resistance of pathogens toward currently available drug therapies is rapidly becoming a major worldwide problem. Thus, the design of new compounds for resistant fungi and bacteria has become one of the most important areas of antibacterial research to date. Plant fungi (e.g., *P. sasakii*, *G. azeae*, *F. oxysporum*, *C. mandshurica*, and *P. infestans*) and bacteria (e.g., *R. solanacearum*) are extremely difficult to control in agricultural production. Pathogenic fungi and bacteria are responsible for billions of dollars in economic losses worldwide each year. In addition, the application of traditional pesticides is not effective and causes high residue level or negative impact on the environment. Therefore, searching for new antifungal and antibacterial agents remains a daunting task in pesticide science. In current study, we combined the active structure of amide and 1, 2, 4-triazole to design and synthesize a series of novel amide derivatives containing a triazole moiety to discover new bioactive molecules and pesticides that can act against fungi and bacteria. Using 2,4-dichloroacetophenone as a starting material, twenty-two novel analogs of amide containing 1,2,4-triazole were synthesized. All the compounds were unequivocally characterized by infrared (IR) spectroscopy, proton and carbon nuclear magnetic resonance spectroscopy (^1^H NMR and^13^C NMR, respectively), and elemental analysis. The biological activity of the compounds against *G. azeae*, *F. oxysporum*, *C. mandshurica*, *P. sasakii*, and *P. infestans* were tested. The results showed that most of the synthesized compounds exhibited antifungal activity against *G. azeae*, *F. oxysporum*, *C. mandshurica*, *P. sasakii*, and *P. infestans* at 50 mg/L and antibacterial activity against *R. solanacearum* at 200 mg/L. Compounds **3e** and **3g** showed high antibacterial activity at 200 mg/L. According to the results of bioassay, compound **4u** displayed higher potent antifungal activity against *P. sasakii* and *G. azeae* than hymexazol. In addition, compounds **4m** and **4q** displayed high antibacterial activity against *R. solanacearum* at 200 mg/L, with 71% and 65% inhibitory rates, respectively. To the best of our knowledge, this study is the first to report on the antibacterial activity of amide derivatives containing a 1,2,4-triazole moiety.

## Results and discussion

### Synthesis

The synthetic route to the title compounds is demonstrated in Additional file 1. Using readily available starting materials, intermediates **1** and **2** were prepared following a previously described procedure [17]. The corresponding acyl chloride was prepared by refluxing acid in thionylchloride for 8 h, and then the solution was diluted with dry dichloromethane at 50°C. The aldol reaction of intermediate **2** with 4-aminobenzaldehyde in dry tetrahydrofuran (THF) using piperidine as a catalyst can proceed readily at 60°C to obtain intermediate **3**. Subsequent treatment of intermediate **3** with acyl chloride and potassium carbonate in dry THF solvent at ambient temperature afforded the desired compounds (**4a** to **4v)** in 40% to 70% yields. The synthesis of compound **4i** was carried out under different conditions to optimize the reaction conditions for the preparation of the title compounds. The effects of different solvents, reaction times, acid binding agents, and reaction temperatures are summarized in Table 1. The yields of compound **4i** were 15.3%, 22.1%, and 40.0% when toluene, acetonitrile, and dichloromethane were used as solvents, respectively (Table 1, Entries 2 to 4). Meanwhile, the yield reached up to 73.8% when the reaction mixture was at 50°C for 8 h in THF (Table 1, Entry 1). The yields of compound **4i** were 33.6%, 55.8.1%, and 73.8% when triethylamine, pyridine, and potassium carbonate were used as acid binding reagents in the reaction, respectively (Table 1, Entries 5 to 7). However, no significant improvement (76.4%, Entry 9) was observed when the reaction time was prolonged from 8 h to 10 h (73.8%, Entry 1).The yield was lower (60.1% after 8 h, Entry 10; 62.2% after10 h, Entry 11) at 25°C than that of 50°C. Hence, the optimum condition was selected in THF with potassium carbonate at 50°C for 8 h. The synthetic route in Scheme 1 has several advantages, including simple procedures, short reaction times, moderate yields, and mild conditions (room temperature).

**Table 1:** Yields of compound 4i at different reaction conditions

**Entry**	**Solvent**	**Time/h**	**Acid binding agent**	**Temperature/°C**	**Yield/%**
1	Tetrahydrofuran	8	Potassium carbonate	50	73.8
2	Toluene	8	Potassium carbonate	50	15.3
3	Acetonitrile	8	Potassium carbonate	50	22.1
4	Dichloromethane	8	Potassium carbonate	50	40.0
5	Tetrahydrofuran	8	Triethylamine	50	33.6
6	Tetrahydrofuran	8	Pyridine	50	55.4
7	Tetrahydrofuran	4	Potassium carbonate	50	62.5
8	Tetrahydrofuran	6	Potassium carbonate	50	67.8
9	Tetrahydrofuran	10	Potassium carbonate	50	76.4
10	Tetrahydrofuran	8	Potassium carbonate	25	60.1
11	Tetrahydrofuran	10	Potassium carbonate	25	62.2

Additional file 2 provides the structure, yield, and elemental analysis data for the title compounds.

The structures of the synthesized compounds were confirmed by elemental analysis and ^1^H-NMR, ^13^C-NMR, and IR spectroscopy. The IR spectral data of compounds **4a** to **4v** showed characteristic absorption bands of NH at 3088 cm^-1^to 3444 cm^-1^. The absorption bands of the carbonyl and C=C groups of *α, β*-unsaturated carbonyl skeleton appeared at 1690 cm^-1^ to 1630 cm^-1^ and 1530 cm^-1^ to 1560 cm^-1^, respectively. In the ^1^H-NMR spectra of the title compounds, most phenyl protons showed multiple at 6.87 ppm to 8.38 ppm. Notably, the phenyl protons of compound **4p** at 9.01 and 9.12 ppm appeared as a singlet because of the existence of two nitro groups in the 3,5-position of the benzene ring, which led to its chemical shift moving to a lower field. The compounds showed the NH proton at 10.53 ppm to 11.07 ppm as a broad singlet. The two protons of the triazole ring appeared at 8.07 ppm to 8.74 ppm and 7.97 ppm to 8.35 ppm. The Ar-OH proton appeared as a broad singlet at 12.07 ppm to 11.44 ppm, and the methyl (Ar-CH_3_) proton signals were observed as a singlet near 2.18 ppm to 2.34 ppm.

### Biological activity and structure-activity relationship (SAR)

#### Antifungal activity

The antifungal bioassay results are shown in Table 2. Hymexazol, one of the commercial fungicides for controlling *G. azeae*, *F. oxysporum*, *C. mandshurica*, *P. sasakii*, and *P. infestans*, was used as the positive control. These newly synthesized amide derivatives containing a triazole moiety exhibited low to high antifungal activities against the tested fungi at 50 mg/L. Compounds **4i**, **4j**, **4k**, and **4u** inhibited the growth of *G. azeae* at 40.06%, 41.22%, 46.44%, 46.01%, and 58.90%, respectively. The activities of compounds **4i**, **4j**, **4k**, **4q**, **4r**, **4u**, and **4v** against *F. oxysporum* were 33.12%, 32.18%, 37.02%, 33.74%, 35.23%, 52.11%, and 30.68%, respectively. And compounds **4i**, **4j**, **4k**, **4q**, **4r**, and **4u** showed 29.41%, 30.11%, 40.01%, 30.65%, 36.77%, 48.61%, and 29.55% activities against *C. mandshurica*, respectively; compounds **4i**, **4j**, **4k**, **4q**, and **4u** displayed activities against *P. sasakii* at 50.06%, 50.22%, 39.68%, 51.25%, and 60.01%, respectively. Moreover, inhibitory rates of compounds **4i**, **4j**, **4k**, **4q**, **4r**, and **4u** on *P. infestans* were 22.52%, 22.18%, 18.82%, 19.04%, 10.33%, and 47.31%, respectively. The preliminary SAR based on the activity against *G. azeae* showed that the substituent group at the 2-position of the benzene ring had an important effect on the antifungal activity of the title compounds. The antifungal activity of the designed compounds decreased when hydroxyl was substituted at the 2-position of the benzene ring, with inhibitory rates ranging from 5.20% to 35.01%. The antifungal activity of compound **4i** (inhibitory rate: 40.44%) without any substituent on the phenyl ring was higher than that of **4a** (inhibitory rate: 29.04%) with hydroxyl at the 2-position of the benzene ring. The variation in substituent on the phenyl ring also caused the different antifungal activities of the title compounds, with inhibitory rates ranging from 0% to 46.44%. For instance, the inhibitory rate of compound **4j** with 2,4-di-fluoro substituent on the phenyl ring was 41.22%, whereas that of compound **4m** with 2,4-di-chloro substituent on the phenyl ring was 31.67%. Furthermore, the compounds with the same substituent but at different positions on the phenyl ring exhibited different antifungal activities. For example, compound **4k** with chorine at the 2-position of the benzene ring possessed high inhibitory activity against *G. azeae*, whereas compound **4l** with chorine at the 4-position of the benzene ring displayed moderate activity. Moreover, when the benzene ring was replaced with a furan ring in the title compounds, compound **4u** showed potent antifungal activity, with inhibitory rates against *G. azeae*, *F. oxysporum*, *C. mandshurica*, and *P. sasakii* ranging from 48.61% to 60.01%. Similar inhibitory rates were exhibited by hymexazol, with 55.54%, 56.12%, 49.61%, and 51.21% against *G. azeae*, *F. oxysporum*, *C. mandshurica*, and *P. sasakii* at 50 mg/L, respectively.

**Table 2:** Antifungal activity of title compounds 4a to 4v at a concentration of 50 mg/L

**Compound**	**Inhibition rate**^**a**^**(%)**				
	***G. zeae***	***F.oxysporum***	***C.mandshurica***	***P.sasakii***	***P. infestans***
**4a**	29.02±0.96	22.91±1.26	24.45±0.74	17.12±0.76	13.96±1.09
**4b**	35.01±0.82	31.26±0.84	13.93±1.86	18.41±0.75	10.56±0.79
**4c**	34.40±0.90	5.88±0.92	21.55±1.32	12.30±0.91	14.88±0.89
**4d**	22.39±0.80	8.66±0.91	16.71±0.82	12.33±0.94	15.61±1.41
**4e**	17.35±1.01	5.26±0.84	8.97±0.72	11.55±1.31	15.26±0.83
**4f**	29.38±0.91	26.38±0.88	20.38±0.61	13.78±0.92	16.33±0.68
**4g**	5.90±1.02	7.84±1.12	12.01±0.88	10.55±1.12	17.82±0.99
**4h**	17.82±1.22	25.75±0.96	21.56±0.91	17.52±0.72	11.53±0.96
**4i**	40.06±0.87	33.12±0.86	29.41±0.83	50.06±0.81	22.52±0.96
**4j**	41.22±1.00	32.18±0.89	30.11±1.33	50.22±1.10	22.18±0.80
**4k**	46.44±0.93	37.02±0.79	40.01±1.17	39.68±0.77	18.82±0.90
**4l**	32.66±0.80	10.12±0.79	18.69±0.93	36.61±0.91	10.32±0.89
**4m**	31.67±0.79	15.68±0.82	19.00±1.34	28.68±0.99	15.64±1.12
**4n**	7.80±1.36	4.65±0.76	24.36±1.02	10.31±0.79	7.14±0.86
**4o**	38.64±0.95	6.52±0.99	27.36±1.44	30.14±0.85	13.02±1.09
**4p**	0	0	12.69±1.00	18.12±0.65	5.10±0.89
**4q**	30.28±0.92	33.74±0.98	30.65±0.95	51.25±0.87	19.04±0.88
**4r**	39.64±0.88	35.23±1.32	36.77±0.75	11.32±0.78	10.33±1.02
**4s**	22.90±1.25	24.36±0.91	26.69±0.89	20.84±1.11	14.33±0.81
**4t**	18.69±1.20	20.13±0.72	15.36±0.90	19.61±0.80	10.13±0.72
**4u**	58.90±0.64	52.11±1.44	48.61±1.03	60.10±0.86	47.31±1.14
**4v**	35.12±0.87	30.68±0.79	29.55±0.94	28.12±0.79	16.88±0.91
Hymexazol^b^	55.54±3.90	56.12±4.10	49.61±7.84	51.21±5.96	68.22±2.41

^a^Average of five replicates, ^b^The commercial agricultural fungicide hymexazol was used for activity comparison.

#### Antibacterial activity

The results of antibacterial bioassay are given in Table 3. Kocide®3000, one of the proven commercial agents for controlling *R. solanacearum*, was used as a reference for bactericides. Most of the prepared compounds showed low to high antibacterial activities against *R. solanacearum* at 200 mg/L. Compounds **4c**, **4n**, and **4s** displayed higher activities than the other compounds at 200 mg/L, reaching 55%, 71%, and 65% inhibitory rates, respectively. The free hydroxyl group plays an important role in the antibacterial activity. Compound **4b** with hydroxyl at the 2-position and chlorine at the 4-position of the benzene ring displayed higher antibacterial activity against *R. solanacearum* than that of compound **4l** with chlorine at the 4-position of the benzene ring. Substituting a methoxy at the 2-position of the benzene ring also improved the antibacterial activity. For example, after introducing a methoxy group into the 2-position of the benzene ring, the inhibitory rate of **4q** (R=2-methoxyphenyl) was 65%. In addition, the inhibitory rates of compounds **4a** (R=2-hydroxyphenyl), **4s** (R=2-fluorophenyl), and **4k** (R=2-chlorophenyl) against tobacco bacterial wilts were 0%, 11%, and 24%, respectively. Furthermore, the activity of the compounds with the same position but with different substituents on the phenyl ring exhibited different activities. For example, the inhibitory rate of compound **4n** with 2,4-dicloro substituent on the phenyl ring was 71%, whereas that of compound **4k** with 2,4-difluro substituent on the phenyl ring was 40%. Unlike antifungal activity, high antibacterial activity was not obtained when the benzene ring was replaced with a heterocyclic ring, i.e., furan ring for compound **4u** and 2-pyridine ring for compound **4v**. Both compounds **4u** and **4v** displayed low antibacterial activity.

**Table 3:** **Antibacterial activity of compounds 4a to 4v against *R. solanacearum***

**Compound**	**Inhibition rate (%)**^**a**^	
	**200 mg/L**	**100 mg/L**
**4a**	0	/
**4b**	55	23
**4c**	0	/
**4d**	0	/
**4e**	30	28
**4f**	14	6
**4g**	0	/
**4h**	17	0
**4i**	0	/
**4j**	40	0
**4k**	24	0
**4l**	17	10
**4m**	71	29
**4n**	11	6
**4o**	38	5
**4p**	0	/
**4q**	65	26
**4r**	16	6
**4s**	11	14
**4t**	29	21
**4u**	0	/
**4v**	22	1
Kocide3000 [Cu(OH)_2_]	100.0	100.0

### Experimental

#### Chemistry

Melting points were determined using a XT-4 binocular microscope (Beijing Tech Instrument Co., Beijing, China) and uncorrected. ^1^H and ^13^C-NMR spectra were recorded on a JEOL-ECX 500 NMR spectrometer operating at 500 MHz for ^1^H-NMR and 125 MHz for ^13^ C-NMR at room temperature using DMSO-*d*_6_ as a solvent and tetramethylsilane as an internal standard. IR spectra were recorded in KBr on an IR Pristige-21 spectrometer (Shimadzu Corporation, Japan). Elemental analysis was performed using an Elemental Vario-III CHN analyzer. Analytical thin layer chromatography was performed on silica gel GF254. Unless otherwise stated, all reagents and reactants were purchased from commercial suppliers and were of analytical grade or chemically pure. All anhydrous solvents were dried and purified according to standard techniques before use. 2-Bromo-1-(2,4-dichlorophenyl) ethanone, intermediate **2**, and sodium-1,2,4-triazolide were prepared according to previously reported methods [18,19] and used without further purification (Additional file 3).

#### Antifungal biological assay

The antifungal activity of all synthesized compounds was tested against five pathogenic fungi, *G. azeae*, *F. oxysporum*, *C. mandshurica*, *P. sasakii*, and *P. infestans*, through the poison plate technique [20]. All the compounds were dissolved in dimethyl sulfoxide (DMSO, 10 mL) before mixing with potato dextrose agar (PDA, 90 mL). The compounds were tested at a concentration of 50 mg/L. All fungal species were incubated in PDA at 27 ±1°C for 5 d to obtain new mycelium for antifungal assay. Mycelia dishes approximately 4 mm in diameter were cut from the culture medium. One of the specimens was picked up with a sterilized inoculation needle and then inoculated in the center of the PDA plate aseptically. The inoculated plates were incubated at 27 ±1°C for 5 d. DMSO in sterile distilled water served as the control, whereas hymexazole acted as the positive control. Three replicates were conducted for each treatment. The radial growth of the fungal colonies was measured, and the data were statistically analyzed. The inhibitory effects of the test compounds *in vitro* against these fungi were calculated as follows:

$$I\left(\%\right)=\left[\left(C-T\right)/\left(C-0.4\right)\right]\times 100$$

where C represents the diameter of fungal growth on untreated PDA, T represents the diameter of fungi on treated PDA, and I is the inhibitory rate.

#### Antibacterial Biological Assay

The antibacterial activities of some title compounds against *R. solanacearum* were evaluated via the turbidimeter test [21], with Kocide®3000 as the positive control. The compounds were dissolved in 150 μL DMSO and diluted with water containing Tween-20 (0.1%) to generate final concentrations of 200 and 100 mg/L, which were then added to the toxic nutrient broth (NB) liquid medium in 4 mL tubes. Approximately 80 μL NB liquid medium containing *R. solanacearum* was individually added to the tubes and then shaken at 180 rpm for 48 h at 30°C. The relative inhibitory rate of the circle mycelium compared with a blank assay was calculated as follows:

$$\mathrm{Relative}\;\mathrm{inhibitory}\;\mathrm{rate}\;\left(\%\right)=\left[\left(A_{0}-A_{1}\right)/A_{0}\right]\times 100$$

Where *A*_0_ and *A*_1_represent the corrected optical density values of the control medium of bacilli and the toxic medium, respectively.

## Conclusion

In summary, a series of novel amide derivatives containing a triazole moiety were designed and synthesized through the reaction of intermediate **3** with different acyl chlorides and anhydrous potassium carbonates in THF at room temperature using 2,4-dichloroacetophenone as a starting material. All the prepared compounds were characterized by spectral data (^1^H-NMR, ^13^C-NMR, and IR) and elemental analysis. The fungicidal activities *in vitro* of the compounds against *G. azeae*, *F. oxysporum*, *C. mandshurica*, *P. sasakii*, and *P. infestans* were evaluated. The results showed that the title compounds possessed low to high antifungal activities against the tested fungi. Compound **4u** displayed high antifungal activity. Furthermore, the antibacterial tests indicated that some of the synthesized compounds also possessed moderate activity against *R. solanacearum*. Compounds **4b**, **4m**, and **4q** exhibited high inhibitory activity against tobacco bacterial wilts *in vitro*. The results of preliminary SAR study indicated that the fungicidal activity can be decreased by introduction of hydroxyl at the 2-position of the benzene ring, the compound containing a furan displayed higher antifungal activity against different fungi than that of benzene, and the substituent of 2,4-di-fluoro on the phenyl ring can enhance the activities against *G. azeae*. However, unlike antifungal activity, the free hydroxyl group at the 2-position of the benzene plays an important role in the antibacterial activity against *R. solanacearum*, and the compounds containing 2,4-dicloro showed much higher activity than that of 2,4-difluro, and the introduction of heterocyclic ring could decrease the antibacterial activity. Moreover, the methoxy at the 2-position of the benzene ring also improved the antibacterial activity. Further studies are currently underway to establish a definite SAR.

## Competing interests

The authors declare that they have no competing interests.

## Authors’ contributions

The current study is an outcome of the constructive discussion with Profs.BAS, LHJ, DYH, and SY, who offered necessary guidance to RPT and CLM to carry out their synthesis and characterization experiments. Both RPT and CLM were also involved in drafting the manuscript. JY performed the antifungal tests; JW and LHJ carried out the ^1^H NMR and^13^C NMR spectral analyses and elemental analysis. BAS and SB were involved in revising the manuscript. All authors read and approved the final manuscript.

## Supplementary Material

Additional file 1 **Synthetic route to target compounds 4a to 4v.** Synthetic sequence to the novel amide derivatives containing a triazole moiety from intermediate **3**. Click here for file

Additional file 2 **Yield and elemental analysis data for title compounds 4a to 4v.** Description: Structure, yield, and elemental analysis data for title compounds **4a** to **4v**. Click here for file

Additional file 3 **Experimental details and data of the title compounds 4a to 4v.** Experimental procedure, spectroscopic data of intermediate **3**, title compounds **4a** to **4v**, copies of ^1^H NMR, ^13^C NMR, and IR spectroscopy. Click here for file
