# Supplementary material for: Synthesis, antifungal and antibacterial activity for novel amide derivatives containing a triazole moiety
Source: Chem Cent J. 2013 Feb 12;7:30. doi: 10.1186/1752-153X-7-30 (PMC3598687; doi:10.1186/1752-153X-7-30)
Supplement: Additional file 1 — Synthetic route to target compounds 4a to 4v. Synthetic sequence to the novel amide derivatives containing a triazole moiety from intermediate 3. [file 1752-153X-7-30-S1.doc]

Additional file 1

**Synthesis, antifungal and antibacterial activity for novel amide derivatives containing a triazole moiety**

Ruping Tang, Linhong Jin*, Chengli Mou, Juan Yin, Song Bai, Deyu Hu, Jian Wu Song Yang , Baoan Song*

Address: State Key Laboratory Breeding Base of Green Pesticide and Agricultural Bioengineering, Key Laboratory of Green Pesticide and Agricultural Bioengineering, Ministry of Education, Research and Development Center for Fine Chemicals, Guizhou University, Guiyang 550025, China.

**Scheme 1**
