# Supplementary material for: Synthesis, antifungal and antibacterial activity for novel amide derivatives containing a triazole moiety
Source: Chem Cent J. 2013 Feb 12;7:30. doi: 10.1186/1752-153X-7-30 (PMC3598687; doi:10.1186/1752-153X-7-30)
Supplement: Additional file 2 — Yield and elemental analysis data for title compounds 4a to 4v. Description: Structure, yield, and elemental analysis data for title compounds 4a to 4v. [file 1752-153X-7-30-S2.doc]

Additional file 2

**Synthesis and** **Biological Activity of Novel Triazole Amide Derivatives**

Ruping Tang, Linhong Jin*, Chengli Mou, Juan Yin, Song Bai, Deyu Hu, Jian Wu Song Yang, Baoan Song*

Address: State Key Laboratory Breeding Base of Green Pesticide and Agricultural Bioengineering, Key Laboratory of Green Pesticide and Agricultural Bioengineering, Ministry of Education, Research and Development Center for Fine Chemicals, Guizhou University, Guiyang 550025, China.

Author to whom correspondence should be addressed;

Tel.: +86 8513620521; Fax: +86 8513622211.

E-Mail: BAS: basong@gzu.edu.cn

**Table 3. Structure, yield and elemental analysis data for title compounds 4a-v**

| Compound | R | Yield (%) | Elemental Analysis (Calcd./Found) | | |
| --- | --- | --- | --- | --- | --- |
|  |  |  | C | H | N |
| **4a** | 2-hydroxyphenyl | 40.9 | 60.14/60.61 | 3.36/3.85 | 11.69/11.61 |
| **4b** | 4-chloro-2-hydroxyphenyl | 40.3 | 56.11/56.52 | 2.94/3.31 | 10.09/11.05 |
| **4c** | 5-chloro-2-hydroxyphenyl | 57.7 | 56.11/55.66 | 2.94/3.07 | 10.09/11.38 |
| **4d** | 5-bromo-2-hydroxyphenyl | 61.1 | 51.64/51.71 | 2.71/3.01 | 10.04/10.04 |
| **4e** | 5-iodo-2-hydroxyphenyl | 55.6 | 47.63/47.98 | 2.50/2.71 | 9.26/9.51 |
| **4f** | 3-methyl-2-hydroxyphenyl | 47.2 | 60.86/60.50 | 3.68/4.03 | 11.36/11.35 |
| **4g** | 4-methyl-2-hydroxyphenyl | 44.7 | 60.86/61.14 | 3.68/3.56 | 11.36/11.46 |
| **4h** | 5-methyl-2-hydroxyphenyl | 52.9 | 60.86/60.50 | 3.68/3.79 | 11.36/11.50 |
| **4i** | phenyl | 73.8 | 62.22/62.46 | 3.48/3.72 | 12.09/11.94 |
| **4j** | 2,4-difluorophenyl | 66.4 | 57.72/57.78 | 2.95/2.83 | 11.80/11.83 |
| **4k** | 2-chlorophenyl | 68.3 | 57.91/58.28 | 3.04/3.07 | 11.26/11.19 |
| **4l** | 4-chlorophenyl | 61.7 | 57.91/57.72 | 3.04/3.23 | 11.26/11.20 |
| **4m** | 2,4-dichlorophenyl | 62.6 | 54.16/53.89 | 2.65/2.95 | 10.53/10.70 |
| **4n** | 3,5-dimethylphenyl | 69.5 | 63.55/63.52 | 4.10/4.07 | 11.40/10.64 |
| **4o** | 4-nitrophenyl | 67.2 | 56.71/56.92 | 2.97/3.02 | 13.78/13.66 |
| **4p** | 3,5-dinitrophenyl | 70.1 | 52.10/52.42 | 2.55/2.85 | 15.19/15.17 |
| **4q** | 2-methoxyphenyl | 63.8 | 60.86/60.84 | 3.68/3.71 | 11.36/11.50 |
| **4r** | 2-nitro5-chlorophenyl | 65.3 | 53.11/53.62 | 2.60/2.53 | 12.90/13.01 |
| **4s** | 2-fluorophenyl | 69.9 | 59.89/59.66 | 3.14/3.21 | 11.64/11.90 |
| **4t** | 3-nitro-4-chlorophenyl | 71.4 | 53.11/53.02 | 2.60/2.43 | 12.90/13.08 |
| **4u** | 2-furanyl | 56.2 | 58.29/58.85 | 3.11/3.17 | 12.36/12.42 |
| **4v** | 2-pyridineyl | 44.7 | 59.50/59.65 | 3.26/3.25 | 15.08/15.23 |
