# Supplementary material for: Synthesis, antifungal and antibacterial activity for novel amide derivatives containing a triazole moiety
Source: Chem Cent J. 2013 Feb 12;7:30. doi: 10.1186/1752-153X-7-30 (PMC3598687; doi:10.1186/1752-153X-7-30)

Additional file 3

**Synthesis, antifungal and antibacterial activity for novel amide derivatives containing a triazole moiety**

Ruping Tang, Linhong Jin*, Chengli Mou, Juan Yin, Song Bai, Deyu Hu, Jian Wu Song Yang, Baoan Song*

Address: State Key Laboratory Breeding Base of Green Pesticide and Agricultural Bioengineering, Key Laboratory of Green Pesticide and Agricultural Bioengineering, Ministry of Education, Research and Development Center for Fine Chemicals, Guizhou University, Guiyang 550025, China.

Author to whom correspondence should be addressed;

Tel.: +86 8513620521; Fax: +86 8513622211.

E-Mail: RPT: [eziotang@yahoo.cn](mailto:eziotang@yahoo.cn)

LHJ: linhong_j@126.com

CLM: mouchengli_h@163.com

JY: yinjuan2009292018@126.com

SB: yinjuan2009292018@126.com

DYU: yinjuan2009292018@126.com

JW: yinjuan2009292018@126.com

BAS: basong@gzu.edu.cn

**Experimental Procedure**

**General Synthetic Procedure for intermediate 3**

To a well-stirred solution of 1-(2,4-dichlorophenyl)-2-(1*H*-1,2,4-triazol-1-yl)ethan- one (0.25 g, 1.00 mmol), 1.20 mmol of 4-aminobenzaldehyde and 20 ml of dry THF, 3 drops of piperdine were added at room temperature. Then, the mixture was heated to reflux and the reaction was allowed to continue till its completion. The solvent was recrystallized twice from dichloromethane to give the refined product in 55% yield.

(**3**; 0.2 g, 56.5%); M.p. 231~233 ^o^C. ^1^H NMR (500 MHz, CDCl_3_, ppm) *δ*: 8.65 (s, 1H, triazole-H), 8.29 (s, 1H, triazole-H), 7.78 (s , 1H, Ar-H), 7.57 (s, 2H, Ar-H), 7.38 (s, 1H, Ar-H), 6.51-6.40 (m, 4H, Ar-H), 6.41-6.40(m, 2H, NH_2_);^13^C-NMR (125 MHz, DMSO-*d*_6_): *δ* 189.26, 154.44, 153.14, 146.66, 146.46, 137.15, 135.68, 134.71, 131.56, 130.74, 130.06, 128.15, 127.01, 117.93, 114.03. IR (KBr, cm^-1^): *ν* 3170.97, 3115.04, 1708.93, 1587.42, 1558.48, 1485.19, 139.39, 823.60, 775.38, 686.66.

**General procedure for the preparation of 4a-v**

The title compounds **4a-v** was prepared by the same synthetic procedure. The corresponding acyl chloride (1.00 mmol) were added to a solution of intermediate 3 (356 mg, 1.00 mmol) and potassium carbonate (160 mg, 1.20 mmol) in dry tetrahydrofuran (10 mL) under 10 °C, then reacted at 50 ºC for 8 h. The progress of the reaction was monitored by TLC. After the completion of the reaction, the mixture was ﬁltered off and the solvent was evaporated off in vacuum and the residue was purified by chromatography on silica gel (ethyl acetate/petroleum ether=*V*/*V* 1:15) to give the desired compounds **4a**–**v** with moderate yields ranging from 40% to 70%.

**The physical and spectral data for 4a-v are listed below.**

**Data for *N*-(4-(3-(2,4-dichlorophenyl)-3-oxo-2-(1*H*-1,2,4-triazol-1-yl)prop-1-en-1- yl)phenyl)-2-hydroxybenzamide (4a).**

Yellow solid, M.p. 211~213 °C. ^1^H-NMR (500 MHz, DMSO-*d*_6_): *δ* 11.44 (s, 1H, OH), 10.58 (s, 1H, NH), 8.76 (s, 1H, triazole-H), 8.35 (s, 1H, triazole-H), 7.84~7.86 (m, 2H, CH=, Ar-H) , 7.68~7.72 (m, 4H, Ar-H), 7.63 (dd, 1H, ^4^*J*_HH_ *=* 1.75 Hz *,* ^3^*J*_HH_ = 8.00 Hz, Ar-H), 7.41~7.46 (m, 1H, Ar-H), 6.91~7.00 (m, 4H, Ar-H). ^13^C-NMR (125 MHz, DMSO) *δ*: 190.06, 166.77, 158.03, 153.37, 146.55, 144.78, 142.54, 136.38, 136.19, 134.21, 132.59, 131.79, 131.75, 131.17, 130.28, 130.06, 128.28, 126.65, 120.63, 119.76, 118.78, 117.60. IR (KBr, cm^-1^): *ν* 3381.21, 3344.57, 1653.00, 1583.56, 1558.48, 1271.09, 1180.44, 825.33, 779.24, 669.30.

***N*-(4-(3-(2,4-dichlorophenyl)-3-oxo-2-(1*H*-1,2,4-triazol-1-yl)prop-1-en-1-yl)phenyl)-4-chloro-2-hydroxybenzamide (4b) .**

Yellow solid, M.p. >250 ^o^C. ^1^H-NMR (500 MHz, DMSO-*d*_6_): *δ* 11.79 (s, 1H, OH), 10.55 (s, 1H, NH), 8.74 (s, 1H, triazole-H), 8.38 (s, 1H, triazole-H), 7.82~7.84 (m, 2H, CH=, Ar-H), 7.67~7.69 (m, 4H, Ar-H), 7.62 (dd, 1H, ^4^*J*_HH_ = 1.70 Hz, ^3^*J*_HH_ = 8.55 Hz, Ar-H), 7.01~7.04 (m, 2H, Ar-H), 6.93 (s, 1H, Ar-H), 6.91 (s, 1H, Ar-H). ^13^C-NMR (125 MHz, DMSO) *δ*: 190.07, 165.65, 158.49, 153.37, 146.55, 144.78, 144.73, 142.42, 137.82, 136.38, 136.16, 132.60, 131.83, 131.77, 131.17, 130.28, 128.28, 126.71, 120.55, 119.89, 118.82, 117.12. IR (KBr, cm^-1^): *ν* 3128.54, 3086.11, 1670.56, 15412.12, 1521.84, 1319.31, 1273.02, 1041.56, 877.61, 835.18.

***N*-(4-(3-(2,4-dichlorophenyl)-3-oxo-2-(1*H*-1,2,4-triazol-1-yl)prop-1-en-1-yl)phenyl)-5-chloro-2-hydroxybenzamide** **(4c) .**

Yellow solid, M.p. >250 ^o^C. ^1^H-NMR (500 MHz, DMSO-*d*_6_): *δ* 11.53 (s, 1H, OH), 10.59 (s, 1H, NH), 8.74 (s, 1H, triazole-H), 8.38 (s, 1H, triazole-H), 7.84 (d, 1H, *J* = 1.75 Hz, CH=), 7.82 (d, 1H, *J* = 2.85 Hz, Ar-H), 7.67~7.69 (m, 4H, Ar-H), 7.63 (dd, 1H, ^4^*J*_HH_ = 2.70 Hz, ^3^*J*_HH_ = 8.60 Hz, Ar-H), 7.45 (dd, 1H, ^4^*J*_HH_ = 2.30 Hz, ^3^*J*_HH_ = 8.60 Hz, Ar-H), 7.01 (d, 1H, *J* = 9.00 Hz, Ar-H), 6.93 (s, 1H, Ar-H), 6.91 (s, 1H, Ar-H). ^13^C-NMR (125 MHz, DMSO) *δ*: 190.06, 165.26, 156.57, 153.37, 146.55, 144.72, 142.32, 136.39, 136.16, 133.52, 132.58, 131.82, 131.78, 131.16, 130.28, 129.23, 128.28, 126.81, 123.28, 121.13, 120.59, 119.50. IR (KBr, cm^-1^): *ν* 3302.13, 3132.40, 1674.21, 1539.20, 1514.12, 1317.38, 1230.58, 821.68, 715.59.

***N*-(4-(3-(2,4-dichlorophenyl)-3-oxo-2-(1*H*-1,2,4-triazol-1-yl)prop-1-en-1-yl)phenyl)-5-bromo-2-hydroxybenzamide** **(4d) .**

Yellow solid, M.p. >250 ^o^C. ^1^H-NMR (500 MHz, DMSO-*d*_6_): *δ* 11.54 (s, 1H, OH), 10.57 (s, 1H, NH), 8.74 (s, 1H, triazole-H), 8.38 (s, 1H, triazole-H), 7.93 (d, 1H, *J* = 2.30 Hz, Ar-H) , 7.84 (d, 1H, *J* = 1.75 Hz, CH=), 7.67~7.69 (m, 4H, Ar-H), 7.62 (dd, 1H, ^4^*J*_HH_ = 1.75 Hz, ^3^*J*_HH_ =8.05 Hz, Ar-H ), 7.56 (dd, 1H, ^4^*J*_HH_ = 2.30 Hz, ^3^*J*_HH_ = 8.60 Hz, Ar-H), 6.91~6.96 (m, 3H, Ar-H). ^13^C-NMR (125 MHz, DMSO) *δ*: 190.08, 165.18, 156.87, 153.38, 146.55, 144.75, 142.31, 136.39, 136.33, 136.15, 132.58, 130.09, 131.81, 131.77, 131.17, 130.28, 128.29, 126.81, 121.73, 120.58, 119.87, 110.76. IR (KBr, cm^-1^): *v* 3302.13, 3132.40, 1664.57, 1593.20, 1529.55, 1442.75, 1093.64, 821.68, 767.67, 681.31.

***N*-(4-(3-(2,4-dichlorophenyl)-3-oxo-2-(1*H*-1,2,4-triazol-1-yl)prop-1-en-1-yl)phenyl)-5-iodo-2-hydroxybenzamide (4e).**

Yellow solid, M.p. >250 ^o^C. ^1^H-NMR (500 MHz, DMSO-*d*_6_): *δ* 11.53 (s, 1H, OH), 10.55 (s, 1H, NH), 8.74 (s, 1H, triazole-H), 8.35 (s, 1H, triazole-H), 8.07 (s, 1H, Ar-H), 7.83 (s, 1H, CH= ), 7.67~7.70 (m, 5H, Ar-H), 7.62 (d, 1H, *J* = 8.05 Hz, Ar-H), 6.93 (s, 1H, Ar-H), 6.91 (s, 1H, Ar-H), 6.82 (d, 1H, *J* = 8.55 Hz, Ar-H). ^13^C-NMR (125 MHz, DMSO) *δ*: 190.08, 165.22, 157.47, 153.38, 146.55, 144.76, 142.34, 142.03, 137.86, 136.39, 136.20, 136.16, 132.57, 131.80, 131.78, 131.17, 130.28, 128.29, 126.78, 122.15, 120.59, 120.21. IR (KBr, cm^-1^): *v* 3298.28, 3132.40, 1666.50, 1591.27, 1512.19, 1442.75, 1288.45, 1093.64, 819.75, 765.74, 669.30.

***N*-(4-(3-(2,4-dichlorophenyl)-3-oxo-2-(1*H*-1,2,4-triazol-1-yl)prop-1-en-1-yl)phenyl)-3-methyl-2-hydroxybenzamide** **(4f) .**

Yellow solid, M.p. 224~226 °C. ^1^H-NMR (500 MHz, DMSO-*d*_6_): *δ* 12.07 (s, 1H, OH), 10.58 (s, 1H, NH), 8.75 (s, 1H, triazole-H), 8.34 (s, 1H, triazole-H), 7.85 (s, 1H, CH=) , 7.83 (d, 1H, *J* = 2.30 Hz, Ar-H), 7.68~7.71 (m, 4H, Ar-H), 7.63 (dd, 1H, ^4^*J*_HH_ = 1.75 Hz, ^3^*J*_HH_ = 8.05 Hz, Ar-H ), 7.38 (d, 1H, *J* = 7.45 Hz, Ar-H), 6.95 (s, 1H, Ar-H), 6.93 (s, 1H, Ar-H), 6.88 (t, 1H, *J* = 14.90 Hz, Ar-H), 2.18 (s, 3H, CH_3_). ^13^C-NMR (125 MHz, DMSO) *δ*: 190.06, 169.83, 159.46, 153.37, 146.57, 144.64, 141.97, 136.43, 136.12, 135.90, 132.31, 132.02, 131.80, 131.18, 130.28, 128.28, 127.17, 126.80, 126.17, 121.61, 118.75, 115.01, 16.10. IR (KBr, cm^-1^): *v* 3296.35, 3107.32, 1653.00, 1595.13, 1338.60, 1184.29, 1010.70, 835.18, 785.03, 754.17.

***N*-(4-(3-(2,4-dichlorophenyl)-3-oxo-2-(1*H*-1,2,4-triazol-1-yl)prop-1-en-1-yl)phenyl)-4-methyl-2-hydroxybenzamide** **(4g).**

Yellow solid, M.p. 218~220 °C. ^1^H-NMR (500 MHz, DMSO-*d*_6_): *δ* 11.58 (s, 1H, OH), 10.53 (s, 1H, NH), 8.75 (s, 1H, triazole-H), 8.33 (s, 1H, triazole-H), 7.84 (d, 1H, *J* = 1.75 Hz, CH=), 7.80 (d, 1H, *J* = 8.00 Hz, Ar-H), 7.67~7.70 (m, 4H, Ar-H), 7.62 (dd, 1H, ^4^*J*_HH_ = 2.30 Hz, ^3^*J*_HH_ = 8.60 Hz, Ar-H), 6.90~6.92 (m, 2H, Ar-H), 6.77~6.79 (m, 2H, Ar-H), 2.28 (s, 3H, CH_3_). ^13^C-NMR (125 MHz, DMSO) *δ*: 190.08, 166.99, 158.63, 153.38, 146.55, 145.10, 144.82, 142.50, 136.38, 136.18, 132.59, 131.78, 131.72, 131.17, 130.27, 129.88, 128.28, 126.61, 120.81, 120.73, 117.89, 115.39, 21.65. IR (KBr, cm^-1^): *v* 3282.84, 3128.54, 1664.57, 1585.49, 1527.62, 1444.68, 1182.36, 997.20, 829.93, 763.91, 669.30.

***N*-(4-(3-(2,4-dichlorophenyl)-3-oxo-2-(1*H*-1,2,4-triazol-1-yl)prop-1-en-1-yl)phenyl)-5-methyl-2-hydroxybenzamide** **(4h) .**

Yellow solid, M.p. >250 ^o^C. ^1^H-NMR (500 MHz, DMSO-*d*_6_): *δ* 11.23 (s, 1H, OH), 10.57 (s, 1H, NH), 8.74 (s, 1H, triazole-H), 8.38 (s, 1H, triazole-H), 7.83 (d, 1H, *J* = 1.75 Hz, CH=), 7.65~7.70 (m, 5H, Ar-H), 7.62 (dd, 1H, ^4^*J*_HH_ = 2.30 Hz, ^3^*J*_HH_ = 8.60 Hz, Ar-H ), 7.23 (dd, 1H, ^4^*J*_HH_ = 2.30 Hz, ^3^*J*_HH_ = 8.60 Hz, Ar-H), 6.87~6.92 (m, 3H, Ar-H), 2.25 (s, 3H, CH_3_). ^13^C-NMR (125 MHz, DMSO) *δ*: 190.08, 166.75, 155.87, 153.38, 146.55, 144.82, 142.56, 136.38, 136.17, 134.92, 132.60, 131.77, 131.71, 131.16, 130.27, 129.91, 128.46, 128.28, 126.60, 120.60, 118.40, 117.49, 20.54. IR (KBr, cm^-1^): *v* 3302.13, 3116.97, 1662.64, 1589.34, 1533.41, 1340.53, 1186.22, 1091.71, 875.68, 731.11.

***N*-(4-(3-(2,4-dichlorophenyl)-3-oxo-2-(1*H*-1,2,4-triazol-1-yl)prop-1-en-1-yl)phenyl)benzamide (4i) .**

Yellow solid, M.p. 180~182 °C. ^1^H-NMR (500 MHz, DMSO-*d*_6_): *δ* 10.52 (s, 1H, NH), 8.75 (s, 1H, triazole-H), 8.34 (s, 1H, triazole-H), 7.92 (s, 1H, Ar-H), 7.90 (d, 1H, *J* = 1.70 Hz, Ar-H), 7.84 (d, 1H, *J* = 1.70 Hz, CH=), 7.76 (s, 1H, Ar-H), 7.75 (s, 1H, Ar-H), 7.66~7.69 (m, 2H, Ar-H), 7.59~7.63 (m, 2H, Ar-H), 7.52~7.55 (m, 2H, Ar-H), 6.92 (s, 1H, Ar-H), 6.90 (s, 1H, Ar-H). ^13^C-NMR (125 MHz, DMSO) *δ*: 190.07, 166.59, 153.38, 146.56, 144.90, 143.37., 136.37, 136.19, 134.98, 132.53, 131.77, 131.63, 131.15, 130.27, 129.02, 128.32, 126.37, 120.35. IR (KBr, cm^-1^): *v* 3375.43, 3088.03, 1666.50, 1585.49, 1519.91, 1323.17, 1039.64, 829.39, 763.21.

***N*-(4-(3-(2,4-dichlorophenyl)-3-oxo-2-(1*H*-1,2,4-triazol-1-yl)prop-1-en-1-yl)phenyl)-2,4-difluorobenzamide** **(4j) .**

Yellow solid, M.p. 89~91 °C. ^1^H-NMR (500 MHz, DMSO-*d*_6_): *δ* 10.75 (s, 1H, NH), 8.75 (s, 1H, triazole-H), 8.34 (s, 1H, triazole-H), 7.84 (s, 1H, CH=) , 7.62~7.76 (m, 6H, Ar-H), 7.43~7.47 (m, 1H, Ar-H), 7.22~7.26 (m, 1H, Ar-H), 6.92~6.93 (m, 2H, Ar-H). ^13^C-NMR (125 MHz, DMSO) *δ*: 190.07, 162.89, 153.38, 146.54, 144.71, 142.72., 136.39, 136.15, 132.63, 132.28, 131.81, 131.78, 131.16, 130.28, 128.28, 126.72, 119.93, 112.60, 112.43, 105.52, 105.31, 105.10. IR (KBr, cm^-1^): *v* 3444.87, 3136.25, 1680.00, 1593.20, 1527.62, 1325.10, 1182.36, 1002.98, 846.75, 673.16.

***N*-(4-(3-(2,4-dichlorophenyl)-3-oxo-2-(1*H*-1,2,4-triazol-1-yl)prop-1-en-1-yl)phenyl)2-chlorobenzamide (4k) .**

Yellow solid, M.p. 129~130 °C. ^1^H-NMR (500 MHz, DMSO-*d*_6_): *δ* 10.85 (s, 1H, NH), 8.75 (s, 1H, triazole-H), 8.34 (s, 1H, triazole-H), 7.85 (d, 1H, *J* = 1.70 Hz, CH=), 7.62~7.70 (m, 5H, Ar-H), 7.56~7.59 (m, 2H, Ar-H), 7.50~7.53 (m, 1H, Ar-H), 7.45 (t, 1H, *J* = 14.90 Hz, Ar-H), 6.92 (s, 1H, Ar-H), 6.90 (s, 1H, Ar-H). ^13^C-NMR (125 MHz, DMSO) *δ*: 190.06, 165.90, 153.37, 146.54, 144.72, 142.82., 136.92, 136.39, 136.16, 132.63, 131.98, 131.80, 131.79, 131.16, 130.37, 130.28, 130.25, 129.48, 128.29, 127.87, 126.66, 119.76. IR (KBr, cm^-1^): *v* 3444.87, 3288.63, 1668.43, 1585.49, 1521.84, 1321.24, 1093.64, 875.68, 833.25.

***N*-(4-(3-(2,4-dichlorophenyl)-3-oxo-2-(1*H*-1,2,4-triazol-1-yl)prop-1-en-1-yl)phenyl) 4-chlorobenzamide** **(4l) .**

Yellow solid, M.p. 223~225 °C. ^1^H-NMR (500 MHz, DMSO-*d*_6_): *δ* 10.60 (s, 1H, NH), 8.76 (s, 1H, triazole-H), 8.35 (s, 1H, triazole-H), 7.96 (s, 1H, Ar-H), 7.94 (s, 1H, Ar-H), 7.85 (d, 1H, *J* = 1.70 Hz, CH=), 7.75 (s, 1H, Ar-H), 7.73 (s, 1H, Ar-H), 7.67~7.70 (m, 2H, Ar-H), 7.61~7.64 (m, 3H, Ar-H), 6.91 (s, 1H, Ar-H), 6.92 (s, 1H, Ar-H). ^13^C-NMR (125 MHz, DMSO) *δ*: 190.07, 165.45, 153.38, 146.55, 144.83, 143.14, 137.35, 136.38, 136.18, 133.64, 133.56, 132.25, 131.78, 131.71, 131.15, 130.30, 129.09, 128.27, 126.52, 120.42. IR (KBr, cm^-1^): *v* 3288.63, 3118.90, 1676.14, 1585.49, 1519.91, 1435.04, 1313.52, 1089.78, 829.93, 785.03, 669.30.

***N*-(4-(3-(2,4-dichlorophenyl)-3-oxo-2-(1*H*-1,2,4-triazol-1-yl)prop-1-en-1-yl)phenyl) 2,4-dichlorobenzamide (4m) .**

Yellow solid, M.p. 205~206 °C. ^1^H-NMR (500 MHz, DMSO-*d*_6_): *δ* 10.85 (s, 1H, NH), 8.73 (s, 1H, triazole-H), 8.32 (s, 1H, triazole-H), 7.83 (s, 1H, CH=), 7.77 (s, 1H, Ar-H), 7.62~7.69 (m, 6H, Ar-H), 7.56 (d, 1H, *J* = 8.55 Hz, Ar-H), 6.92 (s, 1H, Ar-H), 6.91 (s, 1H, Ar-H). ^3^C-NMR (125 MHz, DMSO) *δ*: 190.06, 164.99, 153.37, 146.54, 144.66, 142.60, 136.41, 136.13, 135.71, 132.63, 131.87, 131.78, 131.71, 131.17, 130.91, 130.28, 129.81, 128.28, 128.07, 126.81, 119.79. IR (KBr, cm^-1^): *v* 3228.84, 3126.61, 1676.14, 1583.56, 1527.62, 1323.17, 1093.64, 873.75, 831.32, 669.30.

***N*-(4-(3-(2,4-dichlorophenyl)-3-oxo-2-(1*H*-1,2,4-triazol-1-yl)prop-1-en-1-yl)phenyl)-3,5-dimethylbenzamide (4n) .**

Yellow solid, M.p. 253~255 °C. ^1^H-NMR (500 MHz, DMSO-*d*_6_): *δ* 10.46 (s, 1H, NH), 8.75 (s, 1H, triazole-H), 8.34 (s, 1H, triazole-H), 7.84 (d, 1H, *J* = 1.70 Hz, CH=), 7.75 (s, 1H, Ar-H), 7.73 (s, 1H, Ar-H), 7.66~7.69 (m, 2H, Ar-H), 7.63 (dd, 1H, ^4^*J*_HH_ = 1.75 Hz, ^3^*J*_HH_ = 8.05 Hz, Ar-H), 7.23 (s, 1H, Ar-H), 6.90 (s, 1H, Ar-H), 6.89 (s, 1H, Ar-H), 2.34 (s, 6H, CH_3_). ^13^C-NMR (125 MHz, DMSO) *δ*: 190.06, 166.80, 153.37, 146.54, 144.91, 143.47, 138.21, 136.36, 136.20, 135.01, 133.79, 132.53, 131.77, 131.57, 131.14, 130724, 128.28, 126.24, 125.99, 120.28, 21.37. IR (KBr, cm^-1^): *v* 3319.49, 3113.11, 1656.95, 1585.49, 1527.62, 1327.03, 1093.64, 999.13, 831.32, 754.71, 677.01.

***N*-(4-(3-(2,4-dichlorophenyl)-3-oxo-2-(1*H*-1,2,4-triazol-1-yl)prop-1-en-1-yl)phenyl)-4-nitrobenzamide** **(4o) .**

Yellow solid, M.p. 140~142 °C. ^1^H-NMR (500 MHz, DMSO-*d*_6_): *δ* 10.85 (s, 1H, NH), 8.77 (s, 1H, triazole-H), 8.35~8.38 (m, 3H, triazole-H, Ar-H), 8.16 (s, 1H, Ar-H), 8.14 (s, 1H, Ar-H), 7.84 (d, 1H, *J* = 1.70 Hz, CH=), 7.69~7.77 (m, 4H, Ar-H), 7.63 (dd, 1H, ^4^*J*_HH_ = 1.75 Hz, ^3^*J*_HH_ = 8.05 Hz, Ar-H), 6.95 (s, 1H, Ar-H), 6.94 (s, 1H, Ar-H). ^13^C-NMR (125 MHz, DMSO) *δ*:190.08, 164.92, 153.39, 149.82, 146.56, 144.72, 142.78, 140.59, 136.41, 136.14, 132.52, 131.88, 131.79, 131.17, 130.28, 129.89, 128.27, 126.89, 124.14, 120.55. IR (KBr, cm^-1^): *v* 3286.70, 3032.10, 1672.28, 1583.56, 1544.98, 1334.74, 1091.71, 833.25, 759.95, 698.23.

***N*-(4-(3-(2,4-dichlorophenyl)-3-oxo-2-(1*H*-1,2,4-triazol-1-yl)prop-1-en-1-yl)phenyl)-3,5-dinitrobenzamide (4p) .**

Yellow solid, M.p. >250 ^o^C. ^1^H-NMR (500 MHz, DMSO-*d*_6_): *δ* 11.07 (s, 1H, NH), 9.12 (s, 2H, Ar-H), 9.00 (s, 1H, Ar-H), 8.75 (s, 1H, triazole-H), 8.34 (s, 1H, triazole-H), 7.84 (s, 1H, CH=), 7.77 (s, 1H, Ar-H), 7.75 (s, 1H, Ar-H), 7.69~7.70 (m, 2H, Ar-H), 7.63 (d, 1H, *J* = 8.00 Hz, Ar-H), 6.99 (s, 1H, Ar-H), 6.97 (s, 1H, Ar-H). ^13^C-NMR (125 MHz, DMSO) *δ*: 190.09, 162.38, 153.39, 148.62, 146.58, 144.61, 142.30, 137.51, 136.44, 136.11, 132.48, 132.06, 131.79, 131.20, 130.29, 128.70, 128.29, 127.27, 121.94, 120.84, 120.00. IR (KBr, cm^-1^): *v* 3211.12, 3084.18, 1678.07, 1595.13, 1544.98, 1342.46, 1089.78, 844.82, 715.59, 673.16.

***N*-(4-(3-(2,4-dichlorophenyl)-3-oxo-2-(1*H*-1,2,4-triazol-1-yl)prop-1-en-1-yl)phenyl)-2-methoxybenzamide (4q) .**

Yellow solid, M.p. 152~154 °C. ^1^H-NMR (500 MHz, DMSO-*d*_6_): *δ* 10.41 (s, 1H, NH), 8.74 (s, 1H, triazole-H), 8.34 (s, 1H, triazole-H), 7.83 (d, 1H, *J* = 1.70 Hz, CH=), 7.66~7.71 (m, 4H, Ar-H), 7.58~7.63 (m, 2H, Ar-H), 7.51 (t, 1H, *J* = 14.2 Hz, Ar-H), 7.17 (d, 1H, *J* = 8.00 Hz, Ar-H), 7.05 (t, 1H, *J* = 15.45 Hz, Ar-H), 6.90 (s, 1H, Ar-H), 6.88 (s, 1H, Ar-H), 3.87 (s, 3H, OMe). ^13^C-NMR (125 MHz, DMSO) *δ*: 190.06, 165.66, 156.97, 153.36, 146.53, 146.92, 143.11, 136.36, 136.21, 132.91, 132.66, 131.77, 131.57, 131.15, 130.27, 130.12, 128.28, 126.25, 125.07, 121.03, 119.81, 112.52, 56.38. IR (KBr, cm^-1^): *v* 3329.14, 3103.46, 1666.50, 1573.91, 1539.20, 1327.03, 1180.44, 999.13, 833.25, 754.17, 671.23.

***N*-(4-(3-(2,4-dichlorophenyl)-3-oxo-2-(1*H*-1,2,4-triazol-1-yl)prop-1-en-1-yl)phenyl)-2-nitro5-chlorobenzamide (4r) .**

Yellow solid, M.p. 223~225 °C. ^1^H-NMR (500 MHz, DMSO-*d*_6_): *δ* 11.05 (s, 1H, NH), 8.76 (s, 1H, triazole-H), 8.34 (s, 1H, triazole-H), 8.21 (d, 1H, *J* = 8.6 Hz, Ar-H) , 7.98 (s, 1H, Ar-H), 7.87 (d, 1H, *J* = 2.3 Hz, CH=), 7.84~7.85 (m, 1H, Ar-H), 7.60~7.71 (m, 5H, Ar-H), 6.95 (s, 1H, Ar-H), 6.94 (s, 1H, Ar-H). ^13^C NMR (125 MHz, DMSO) *δ*: 190.07, 163.58, 153.38, 146.56, 145.27, 144.63, 142.49, 139.53, 136.42, 136.14, 134.30, 132.65, 131.95, 131.80, 131.57, 131.19, 130.28, 129.71, 128.28, 127.00, 126.95, 119.87. IR (KBr, cm^-1^): *v* 3444.87, 3161.66, 1683.86, 1583.56, 1521.84, 1342.46, 1269.16, 1078.21, 999.13, 833.25.

***N*-(4-(3-(2,4-dichlorophenyl)-3-oxo-2-(1*H*-1,2,4-triazol-1-yl)prop-1-en-1-yl)phenyl)-2-fluorobenzamide (4s) .**

Yellow solid, M.p. 159~161 °C. ^1^H-NMR (500 MHz, DMSO-*d*_6_): *δ* 10.76 (s, 1H, NH), 8.75 (s, 1H, triazole-H), 8.34 (s, 1H, triazole-H), 7.84 (d, 1H, *J* = 1.70 Hz, CH=), 7.59~7.70 (m, 7H, Ar-H), 7.31~7.38 (m, 2H, Ar-H), 6.92 (s, 1H, Ar-H), 6.91 (s, 1H, Ar-H). ^13^C-NMR (125 MHz, DMSO) *δ*: 190.05, 163.79, 153.38, 146.53, 144.74, 142.82, 136.39, 136.16, 133.49, 132.64 131.77, 131.16, 130.46, 130.28, 128.28, 126.64, 125.17, 125.07, 124.95, 119.88, 116.86, 116.69. IR (KBr, cm^-1^): *v* 3404.36, 3109.25, 1674.21, 1587.42, 1525.69, 1325.10, 1093.64, 875.68, 829.39, 750.31.

**4-chloro-*N*-(4-(3-(2,4-dichlorophenyl)-3-oxo-2-(1*H*-1,2,4-triazol-1-yl)prop-1-en-1-yl)phenyl)-3-nitro-4-chlorobenzamide (4t) .**

Yellow solid, M.p. 248~250 °C. ^1^H-NMR (500 MHz, DMSO-*d*_6_): *δ* 10.81 (s, 1H, NH), 8.76 (s, 1H, triazole-H), 8.60 (d, 1H, *J* = 1.70 Hz, Ar-H), 8.35 (s, 1H, triazole-H), 8.21~8.23 (m, 1H, Ar-H), 7.97 (d, 1H, *J* = 8.60 Hz, CH=), 7.85 (d, 1H, *J* = 1.70 Hz, Ar-H), 7.62~7.74 (m, 5H, Ar-H), 6.95 (s, 1H, Ar-H), 6.93 (s, 1H, Ar-H). ^13^C-NMR (125 MHz, DMSO) *δ*: 190.08, 163.48, 153.38, 147.85, 146.57, 144.70, 142.63, 136.41, 136.13, 134.93, 133.50, 132.55, 132.51, 131.90, 131.78, 131.18, 130.28, 128.95, 128.28, 126.94, 125.56, 120.58. IR (KBr, cm^-1^): *v* 3288.63, 3086.11, 1674.21, 1593.20, 1531.48, 1325.10, 1091.71, 835.18, 742.59, 675.09.

***N*-(4-(3-(2,4-dichlorophenyl)-3-oxo-2-(1*H*-1,2,4-triazol-1-yl)prop-1-en-1-yl)phenyl) furan -2-carboxamide (4u) .**

Yellow solid, M.p. 155~157 °C. ^1^H-NMR (500 MHz, DMSO-*d*_6_): *δ* 10.48 (s, 1H, NH), 8.76 (s, 1H, triazole-H), 8.34 (s, 1H, triazole-H), 7.97 (s, 1H, Furan-H), 7.85 (s, 1H, CH=), 7.63~7.74 (m, 5H, Ar-H), 7.37 (s, 1H, Furan-H), 6.90 (s, 1H, Ar-H), 6.89 (s, 1H, Ar-H), 6.71 (s, 1H, Furan-H). ^13^C-NMR (125 MHz, DMSO) *δ*: 190.06, 156.87, 153.39, 147.50, 146.80, 146.55, 142.81, 136.37, 136.18, 132.53, 131.77, 131.64, 131.15, 130.27, 128.27, 126.40, 120.53, 116.22, 112.91. IR (KBr, cm^-1^): *v* 3228.84, 3109.25, 1662.64, 1585.49, 1514.12, 1271.09, 1093.64, 829.39, 781.17, 673.16.

***N*-(4-(3-(2,4-dichlorophenyl)-3-oxo-2-(1*H*-1,2,4-triazol-1-yl)prop-1-en-1-yl)phenyl)picolinamide (4v) .**

Yellow solid, M.p. 189~191 °C. ^1^H-NMR (500 MHz, DMSO-*d*_6_): *δ* 10.96 (s, 1H, NH), 8.78 (s, 1H, triazole-H), 8.75 (d, 1H, *J* = 4.6 Hz, pyridine-H), 8.34 (s, 1H, triazole-H), 8.15 (d, 1H, *J* = 8 Hz, pyridine-H), 8.07 (t, 1H, *J* = 14.9 Hz, pyridine-H), 7.82~7.93 (m, 3H, pyridine-H, Ar-H) , 7.62~7.69 (m, 4H, Ar-H), 6.93 (s, 1H, Ar-H), 6.92 (s, 1H, Ar-H). ^13^C-NMR (125 MHz, DMSO) *δ*: 190.08, 163.58, 153.42, 149.92, 149.04, 146.58, 144.79, 142.53, 138.79, 136.39, 136.17, 132.52, 131.78, 131.70, 131.16, 130.28, 128.27, 127.81, 126.65, 123.21, 120.56. IR (KBr, cm^-1^): *v* 3329.14, 3109.25, 1683.86, 1575.84, 1525.69, 1411.89, 1286.52, 1078.21, 827.46, 746.45, 675.09.

**IR, ^1^H**-**NMR and ^13^C**-**NMR of intermediate 3 and 4a-v**


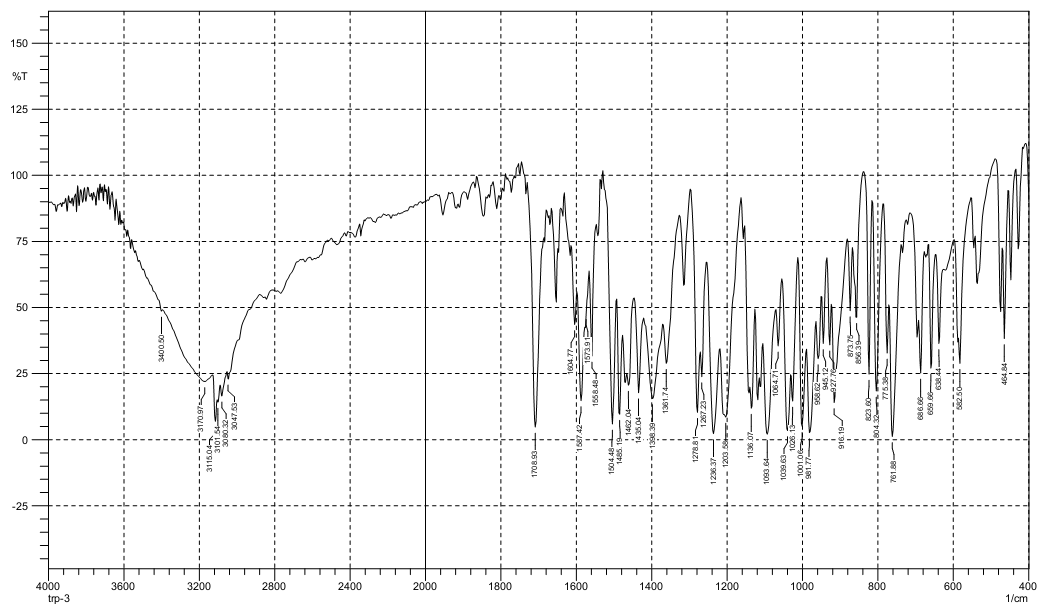


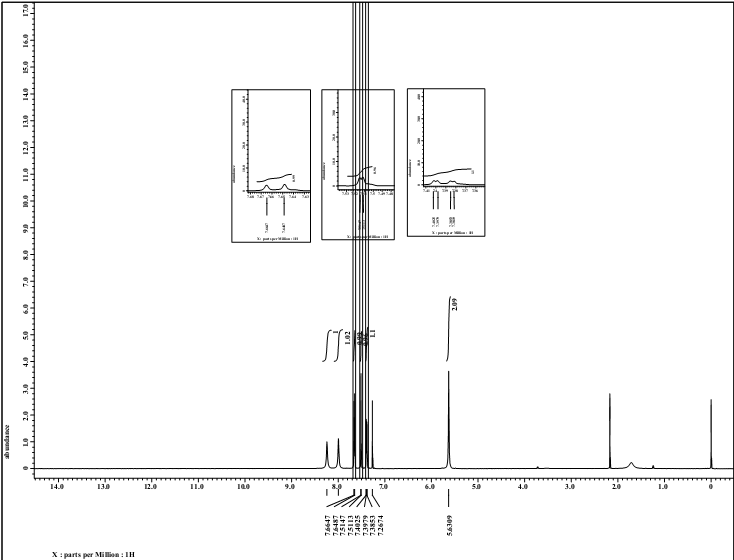


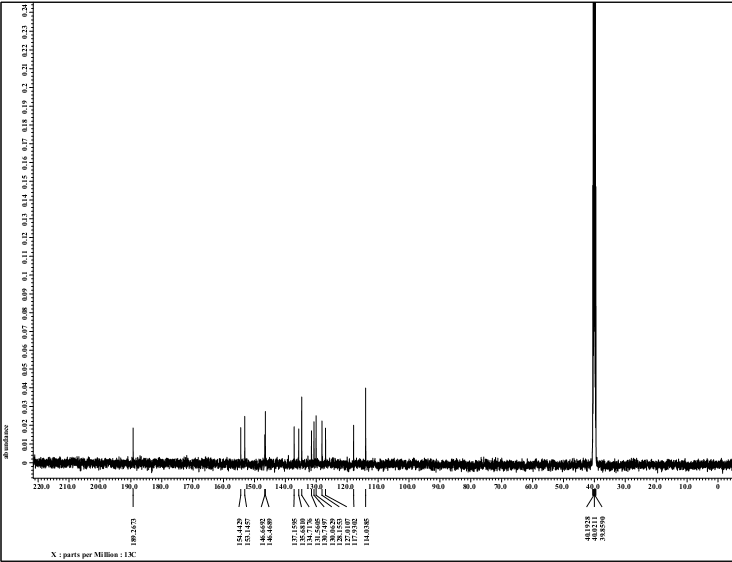


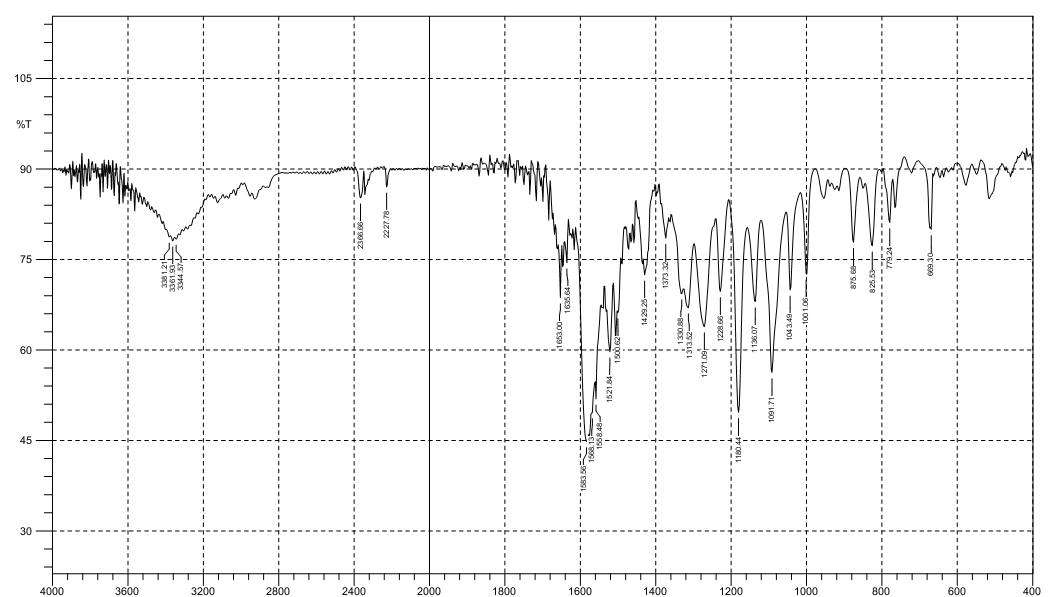


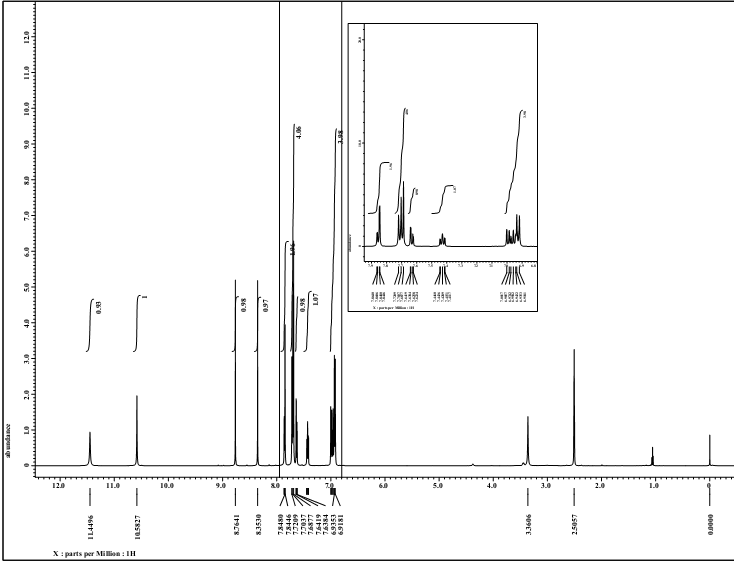

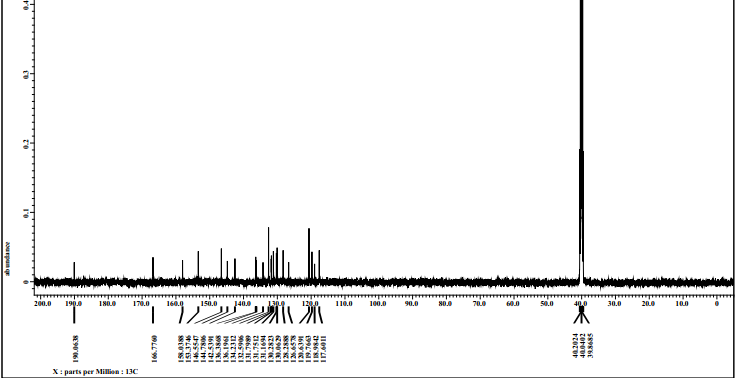


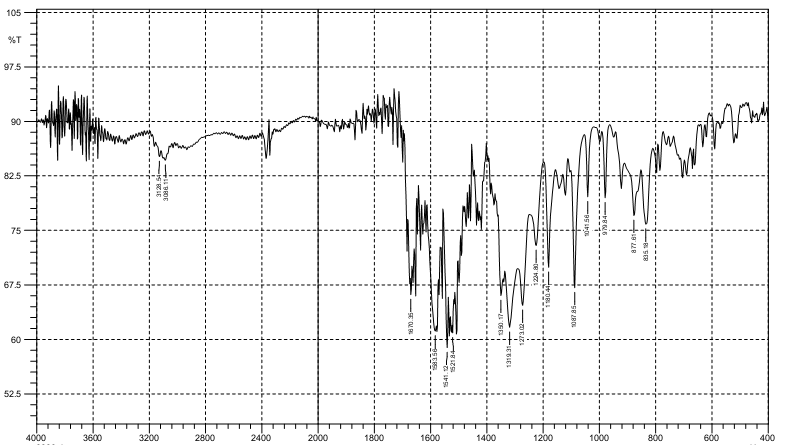


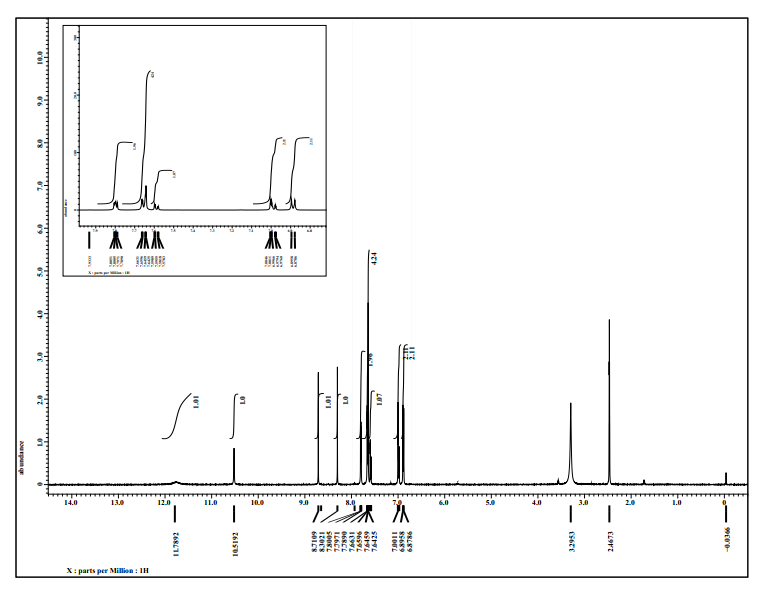

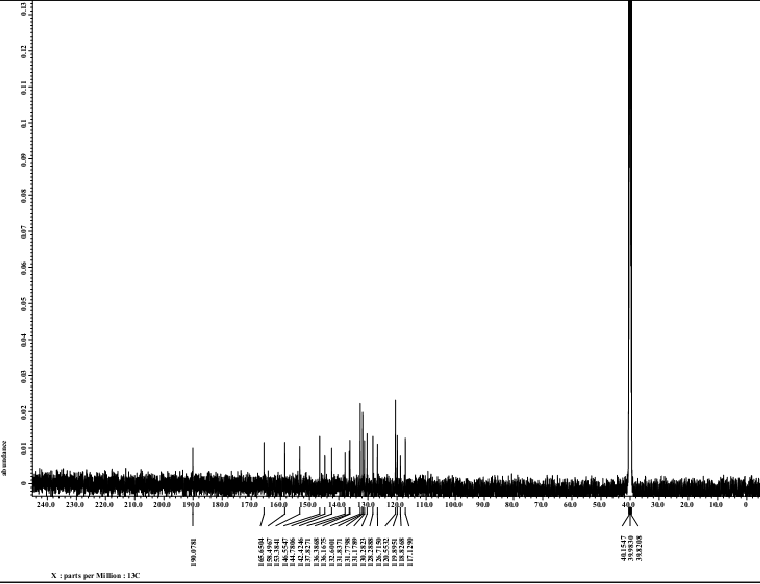


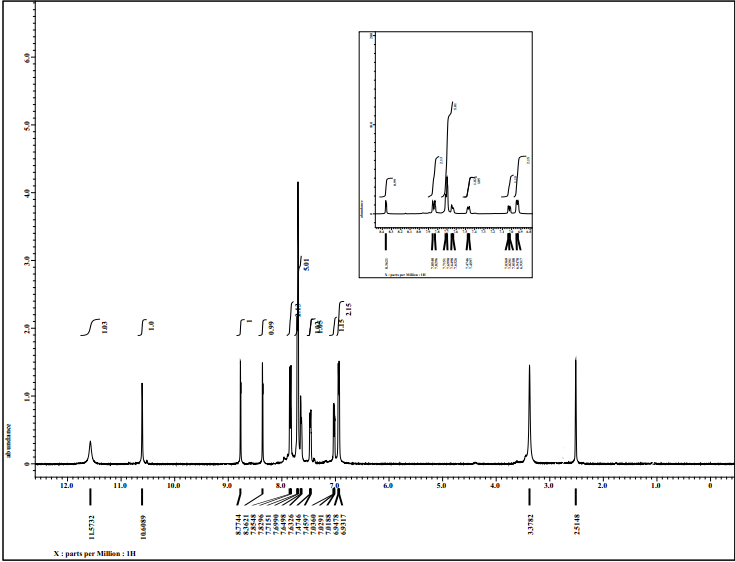

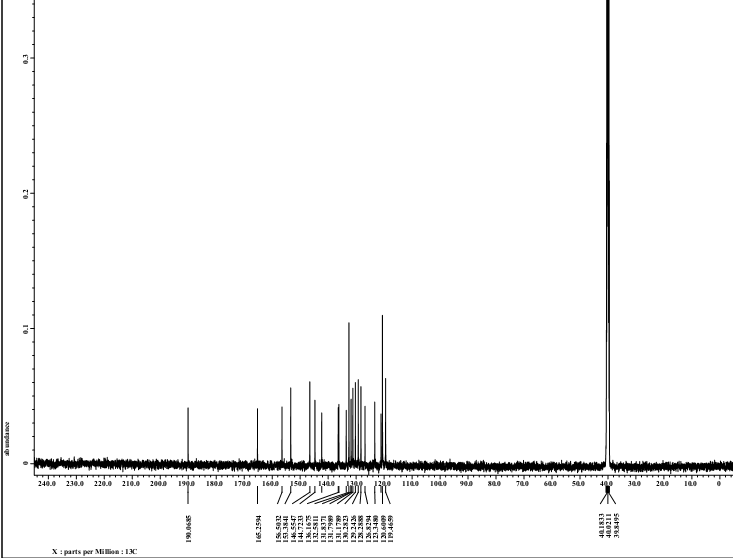

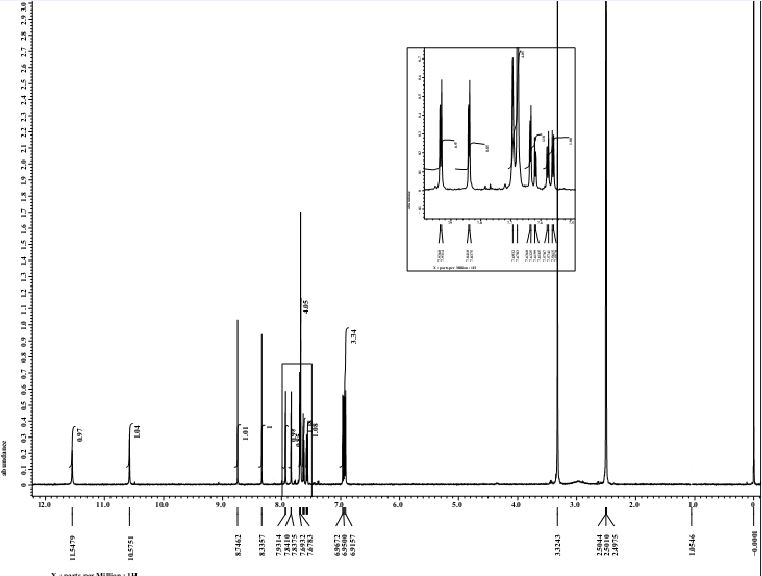

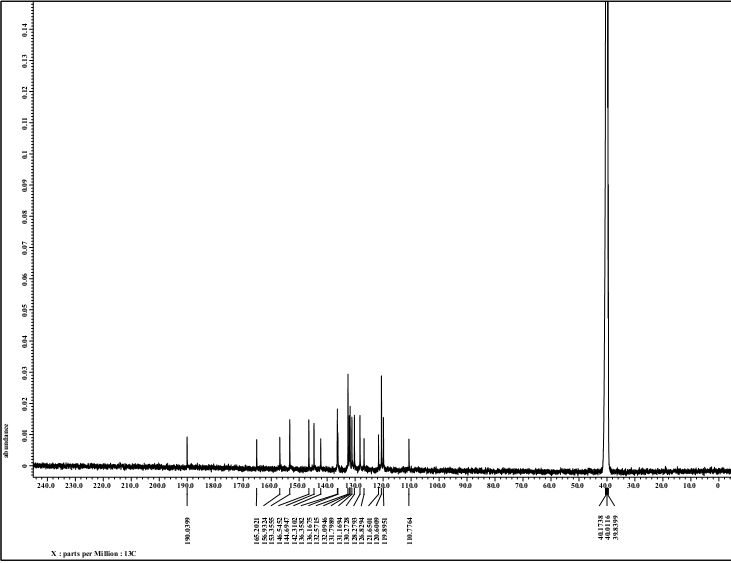


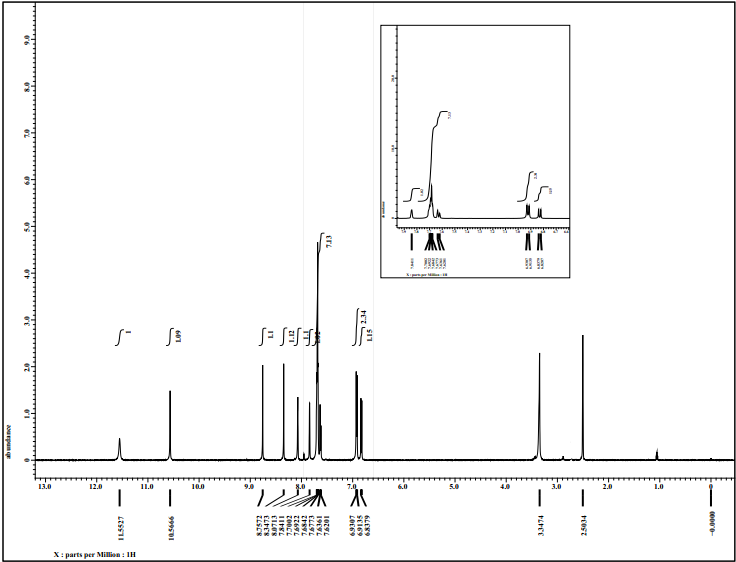

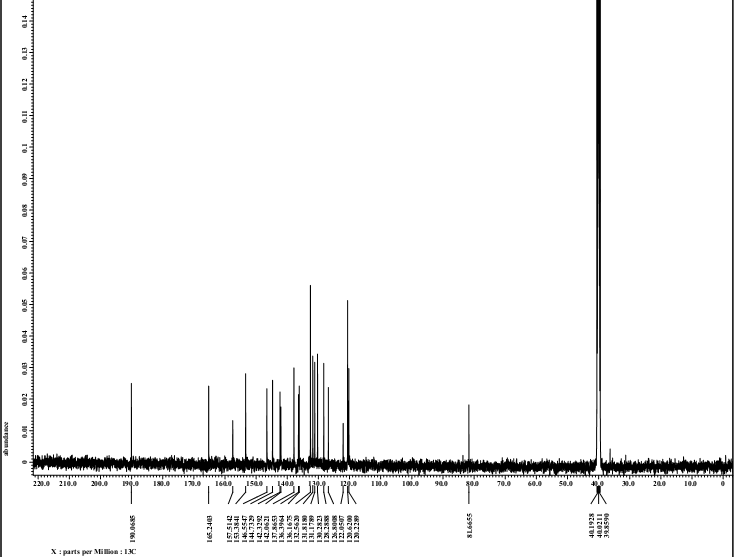

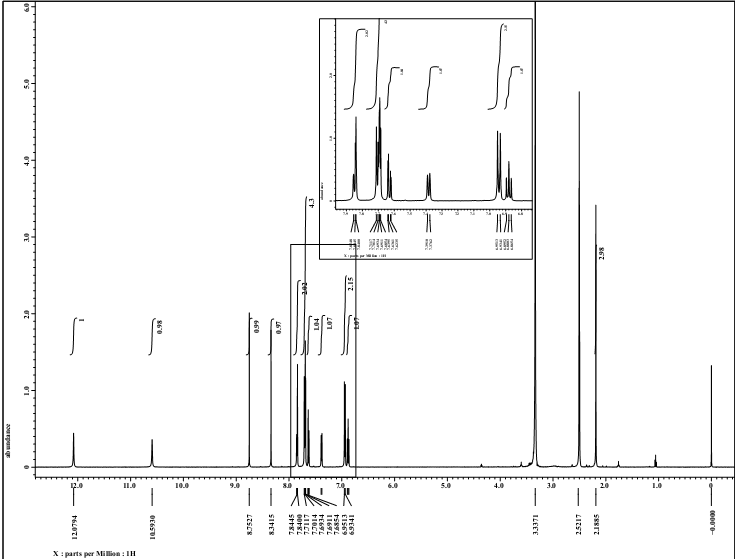

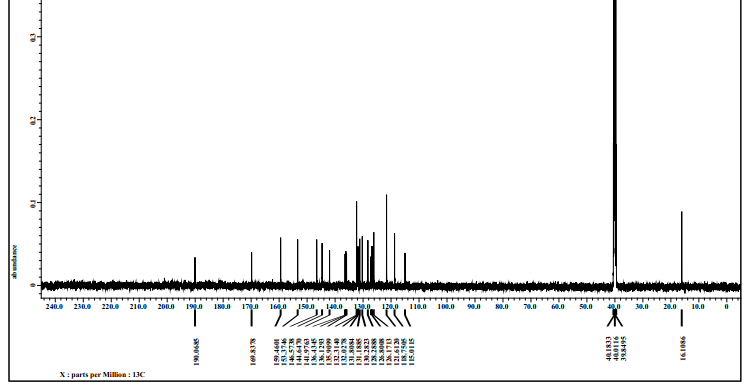

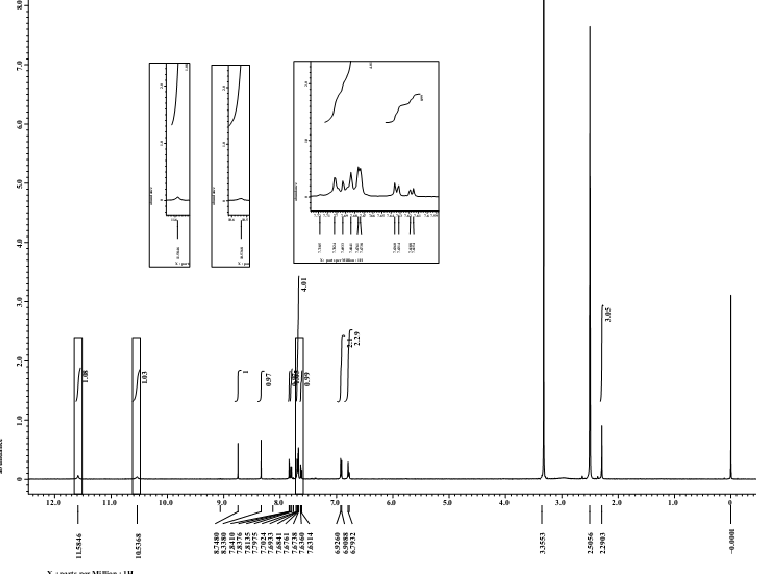

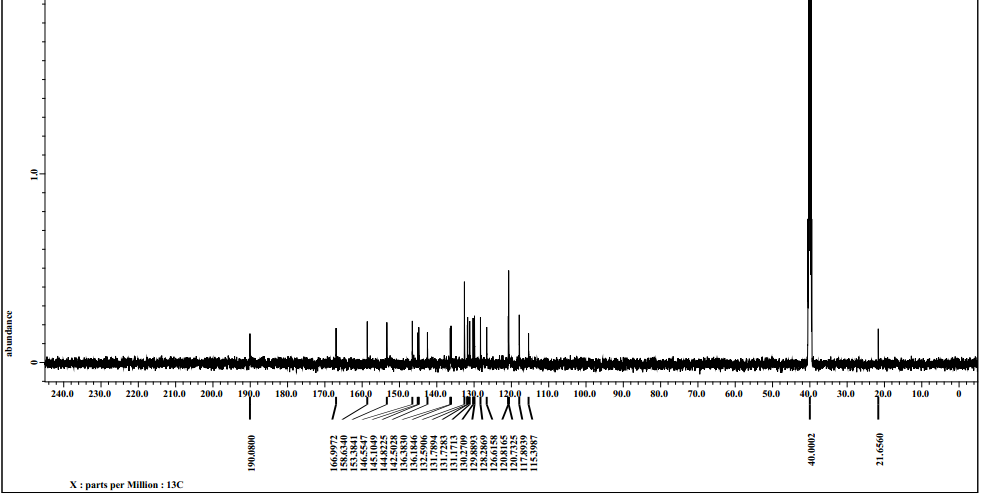

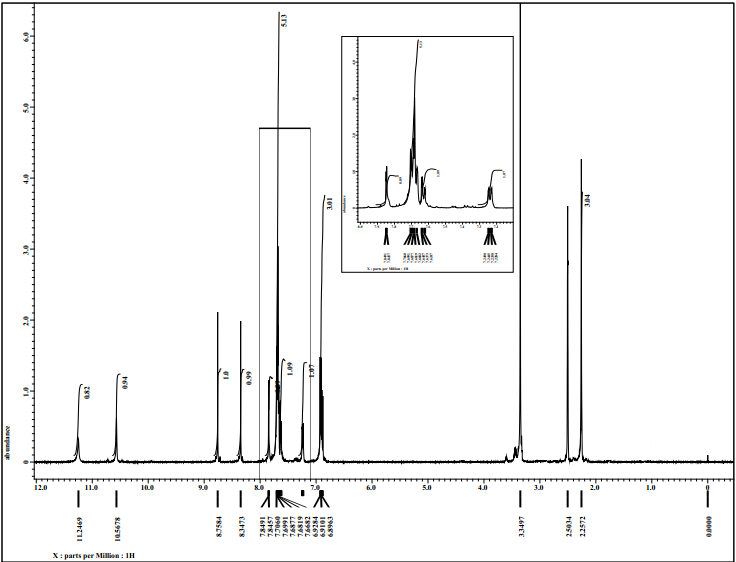

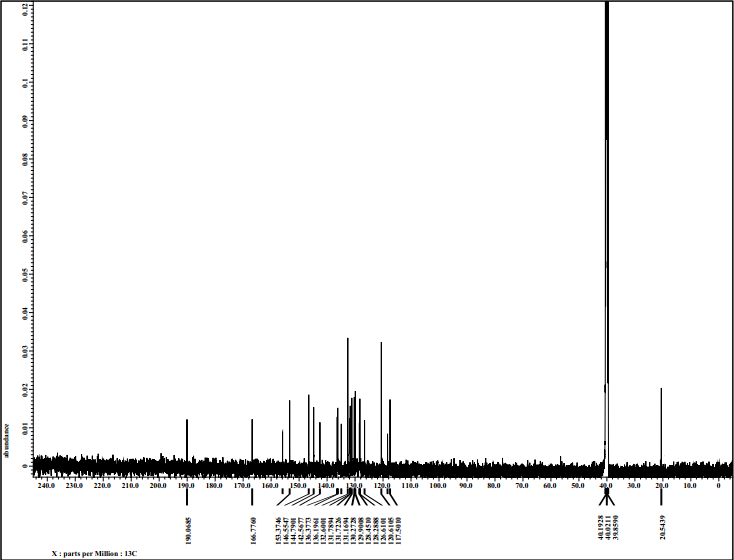


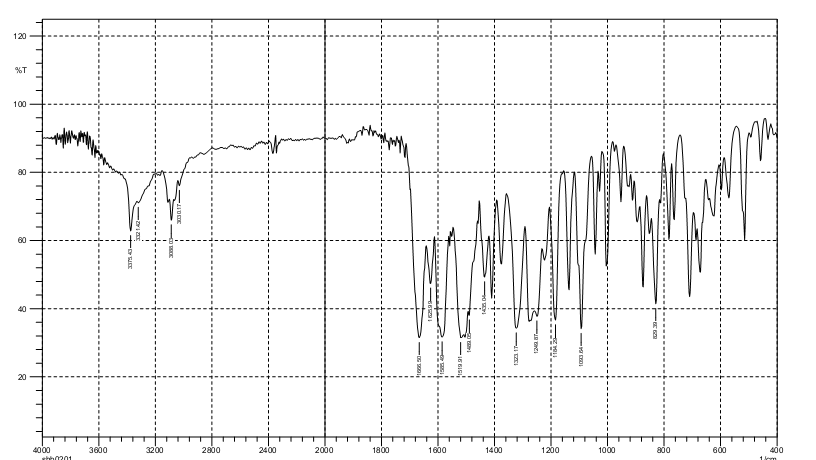

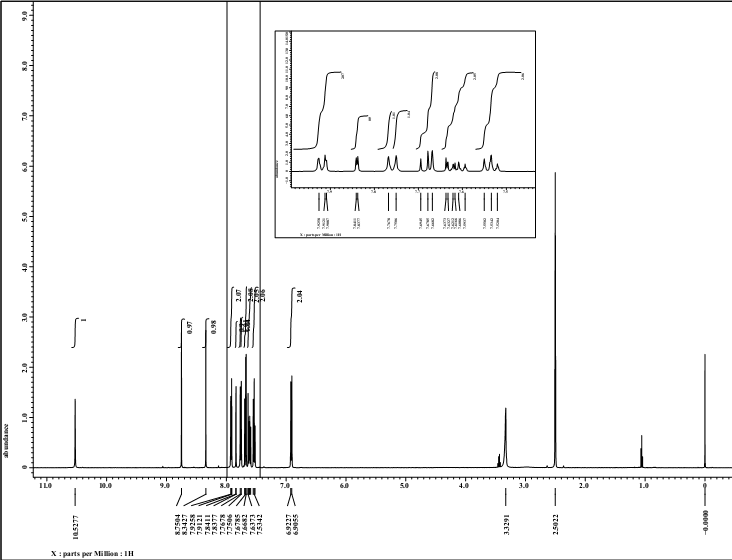

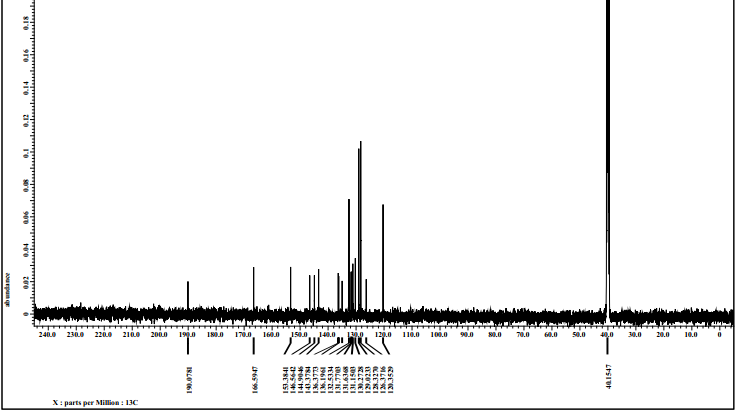

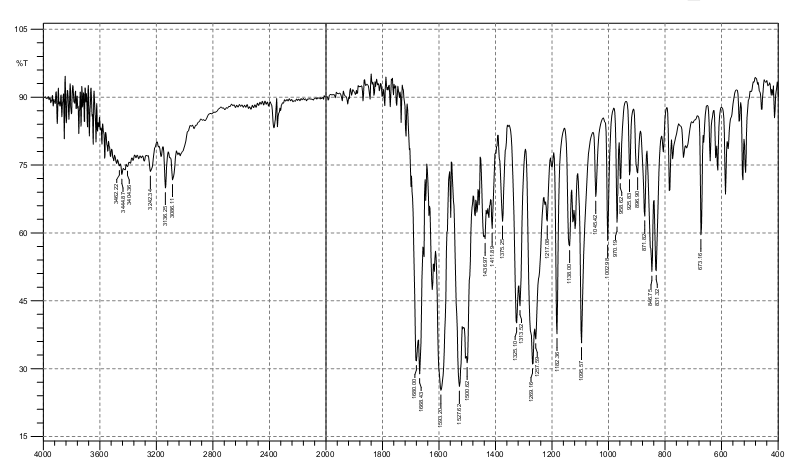

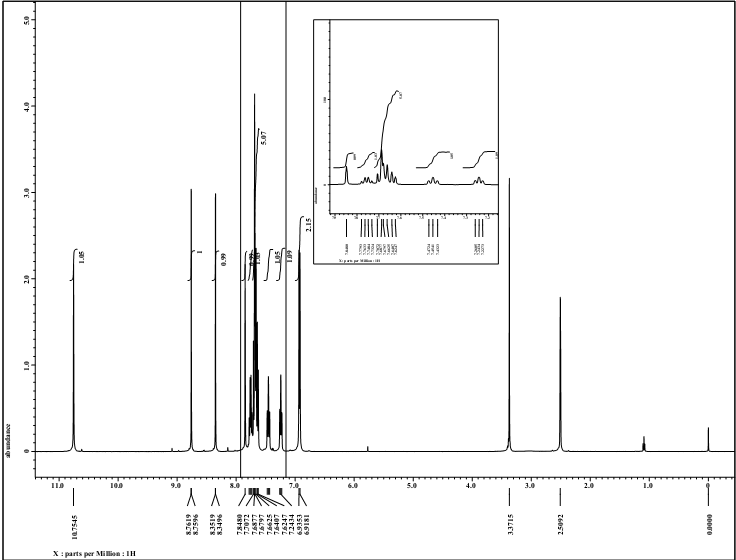

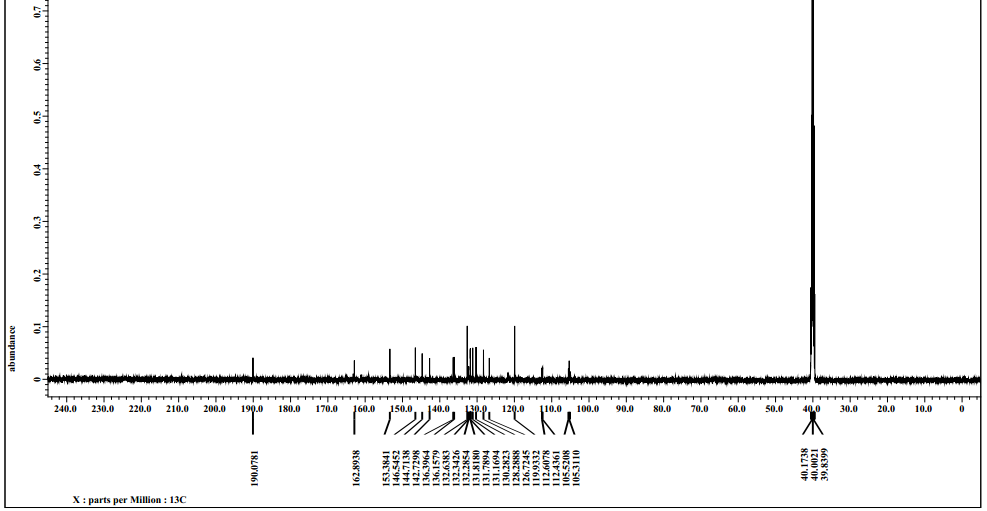

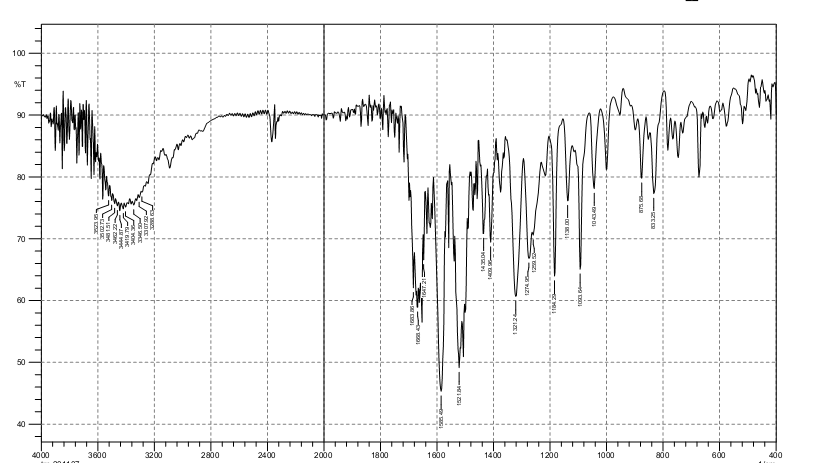

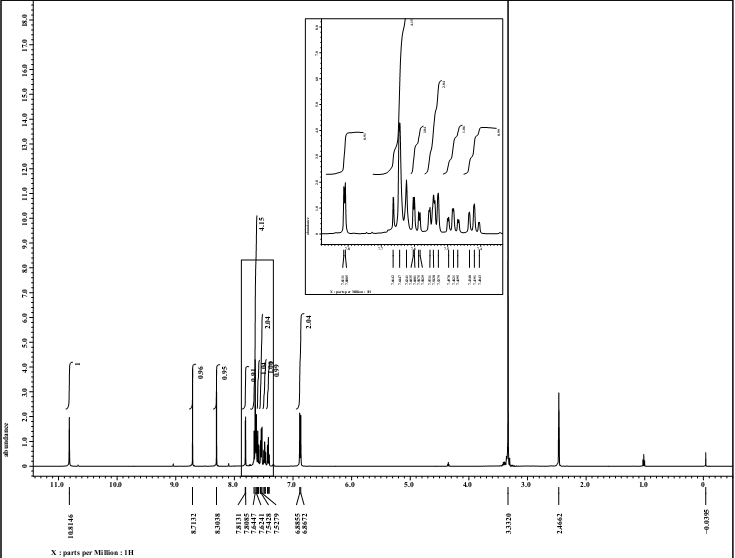

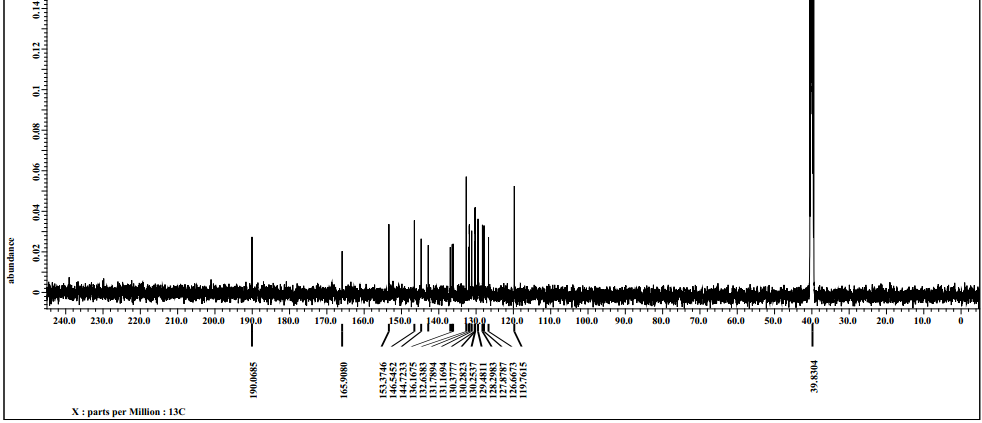

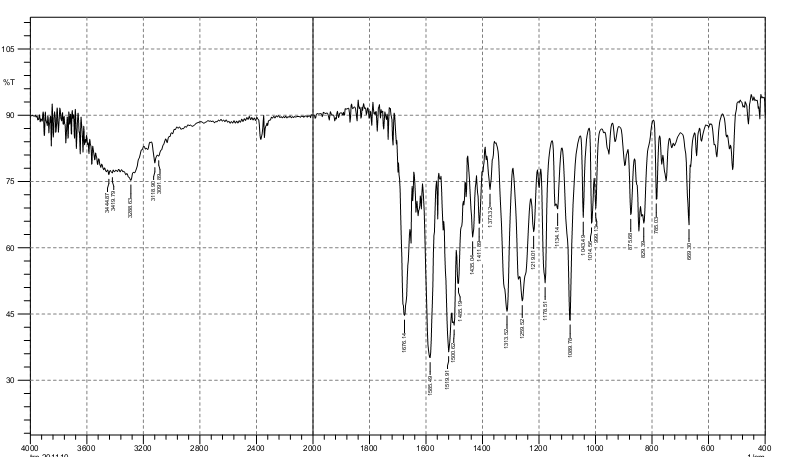

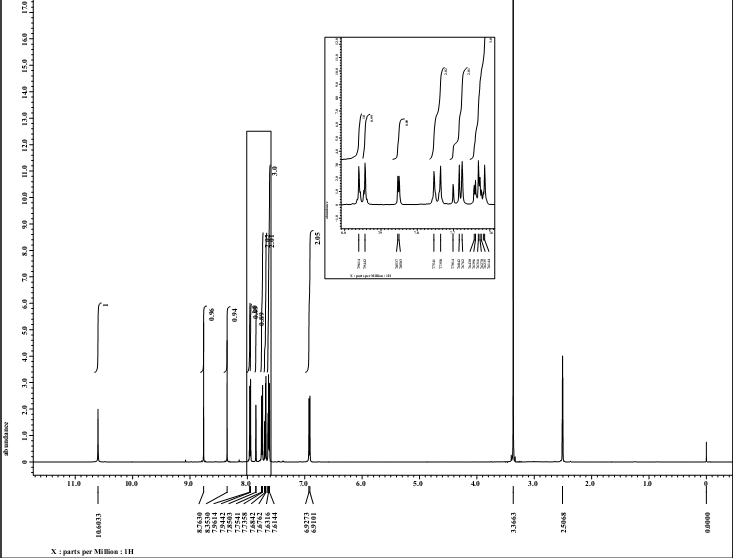

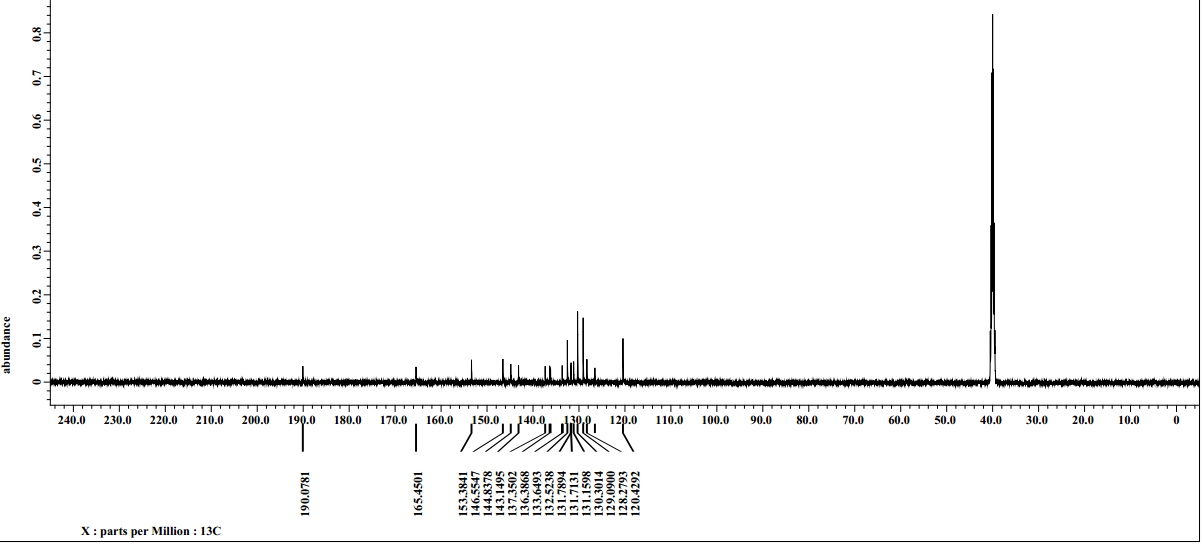

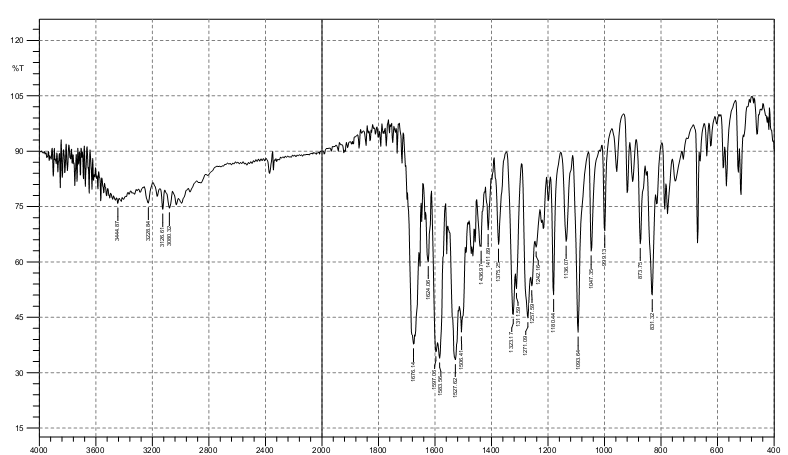

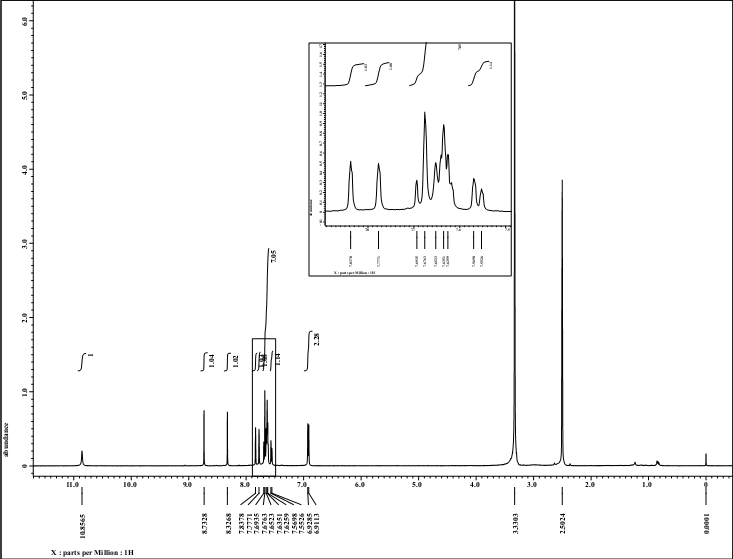

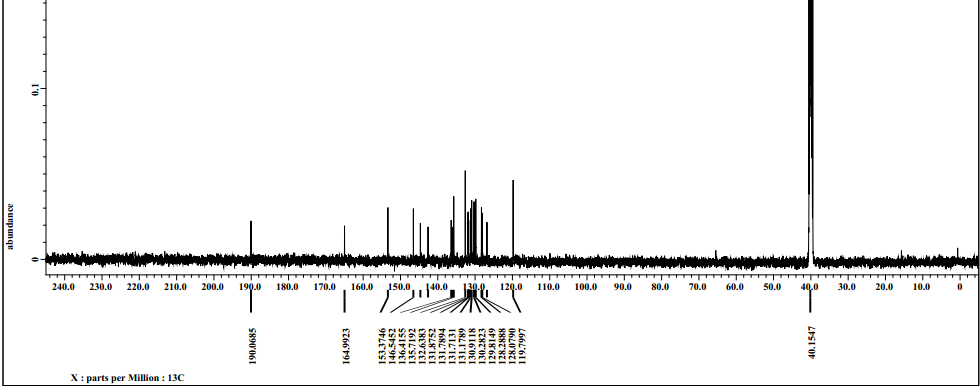

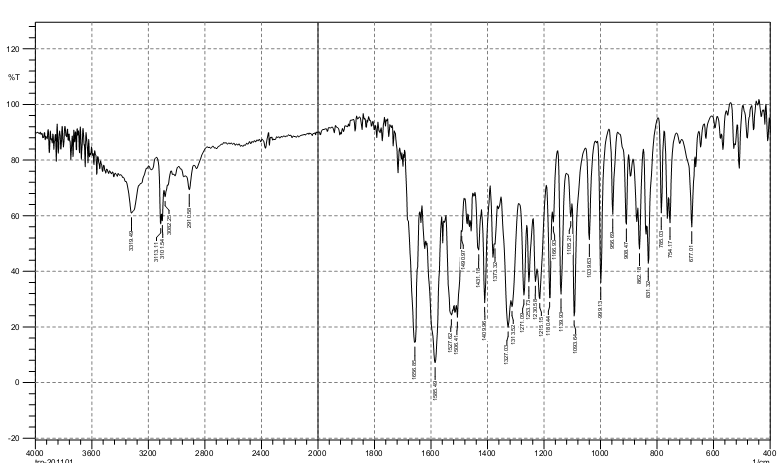

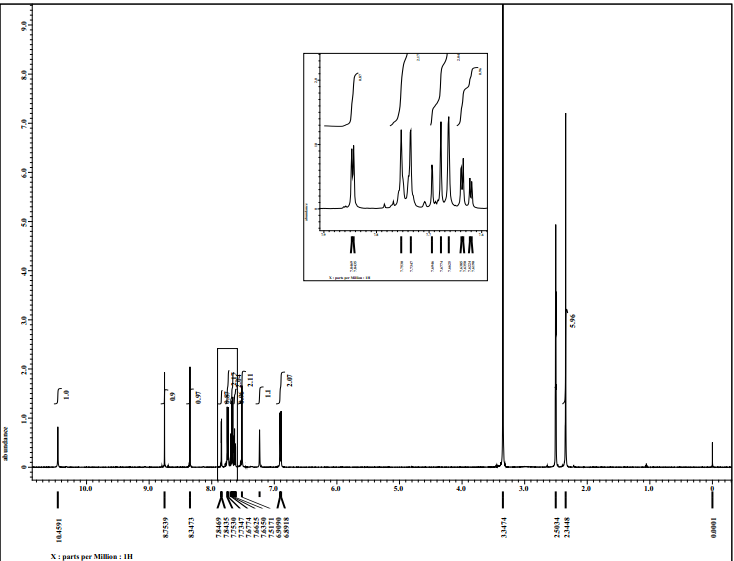

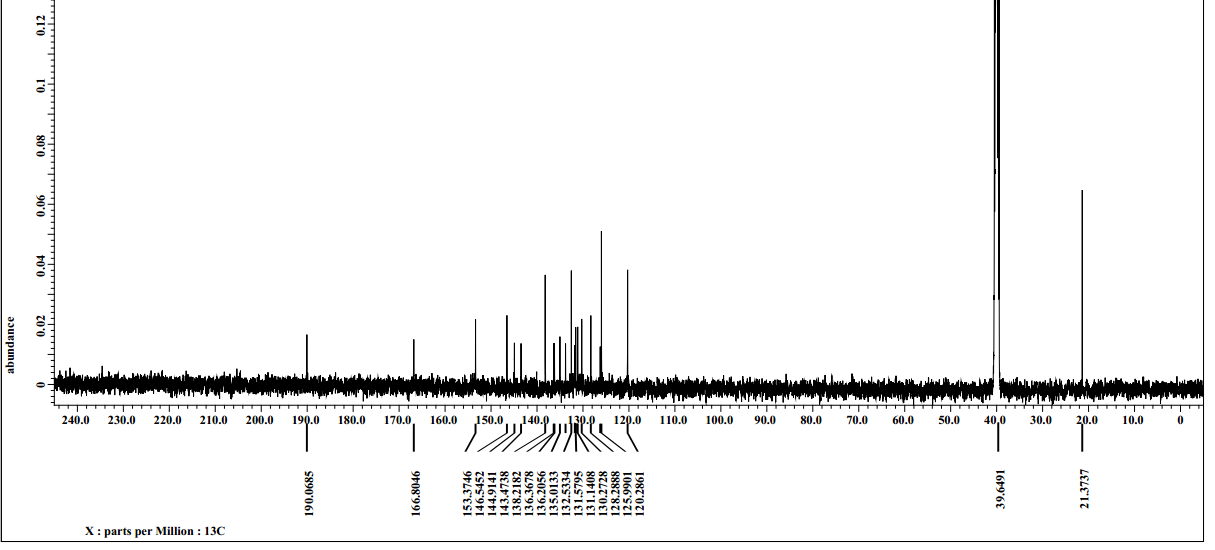

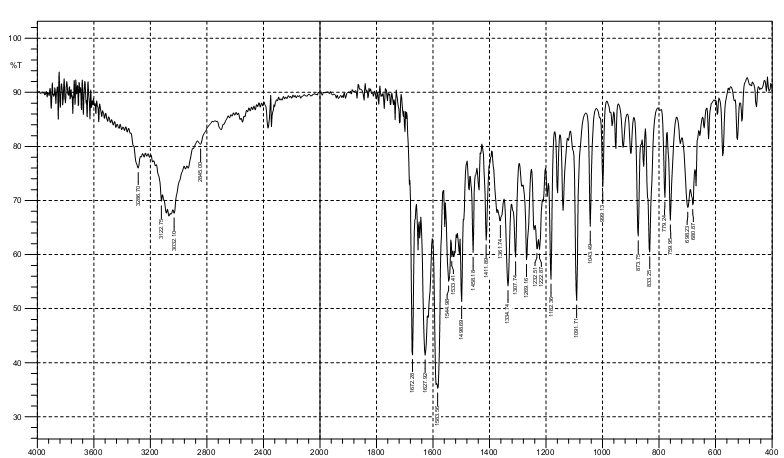

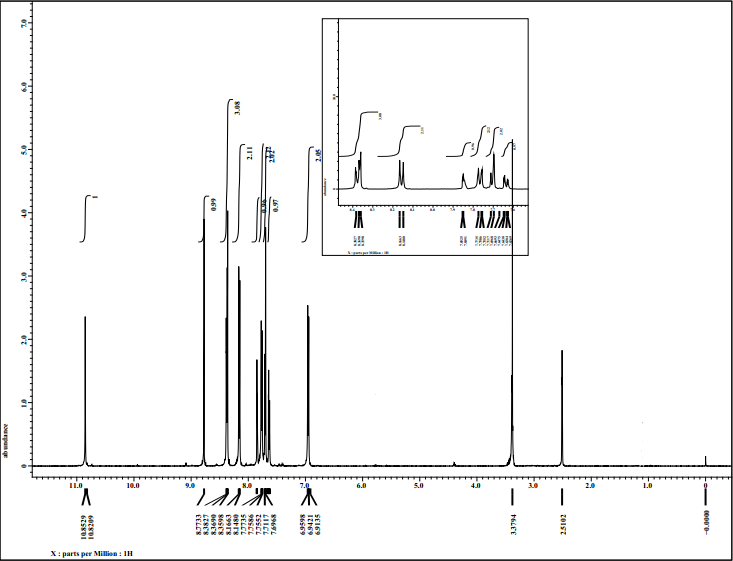

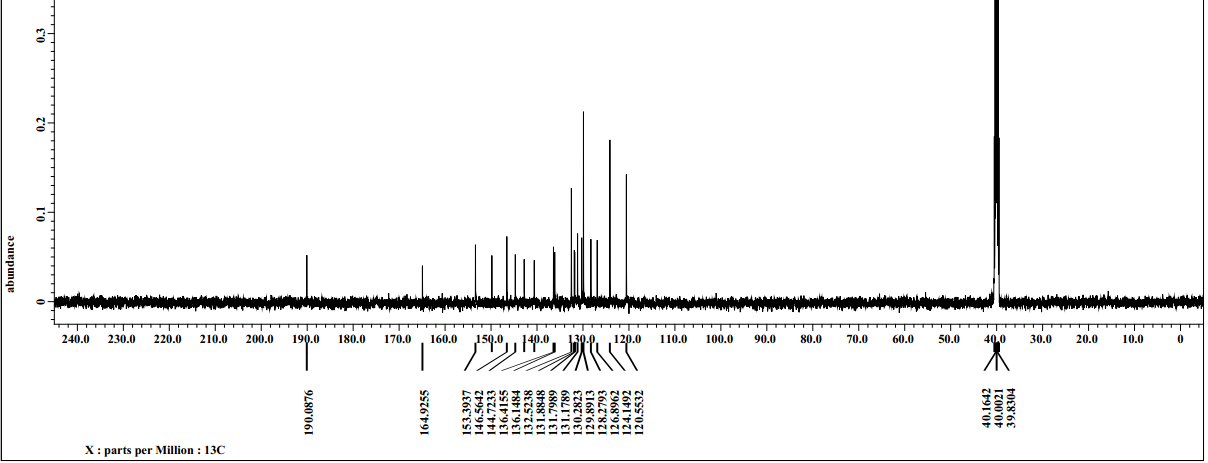

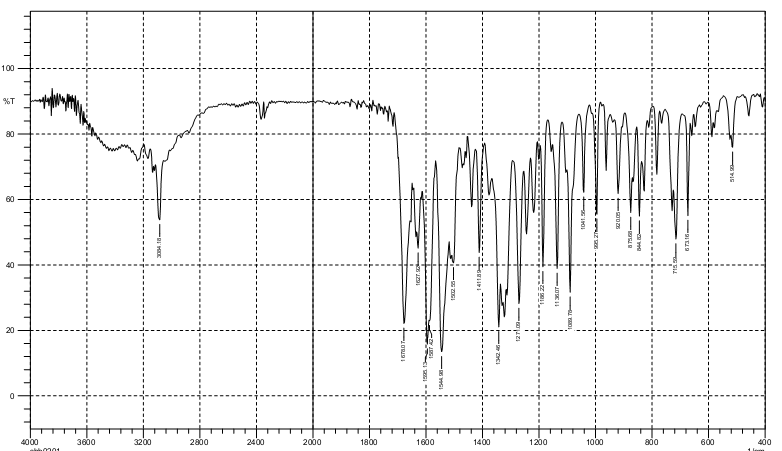

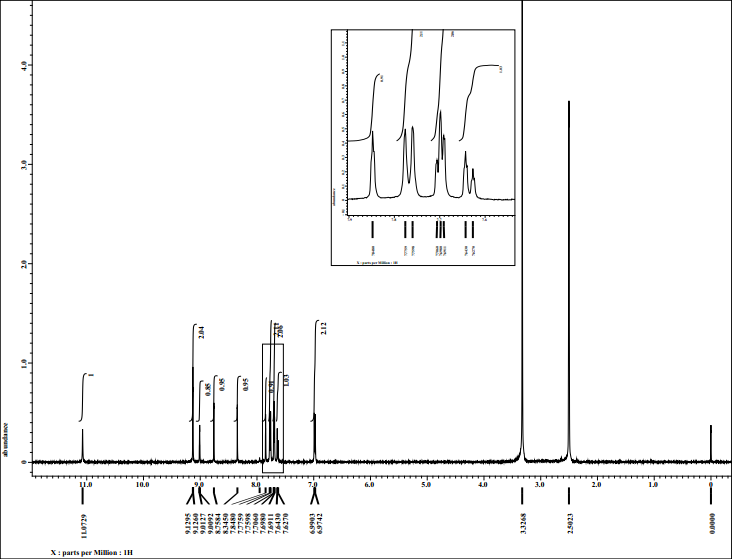

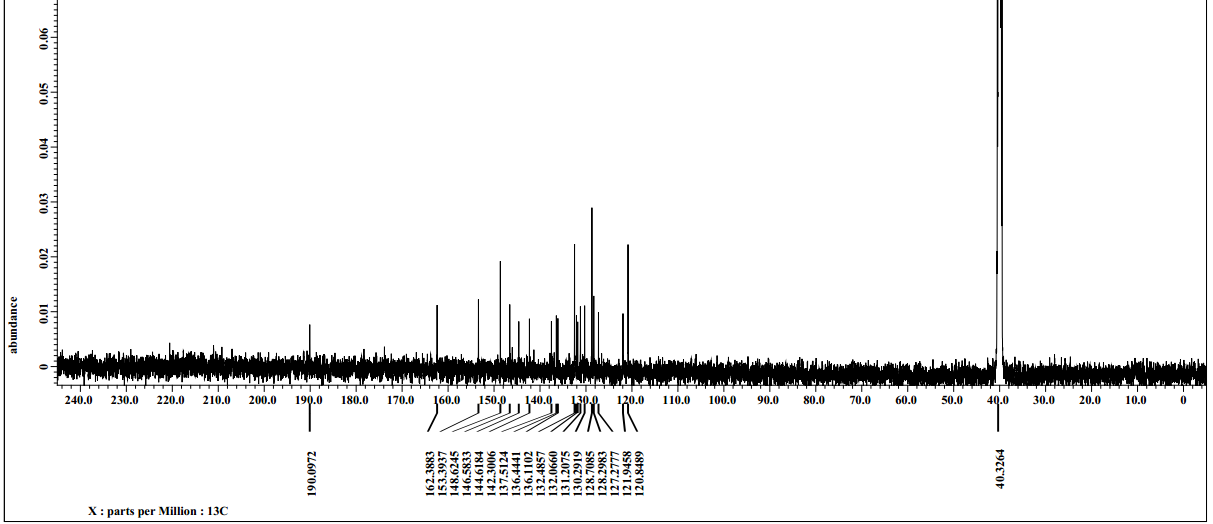

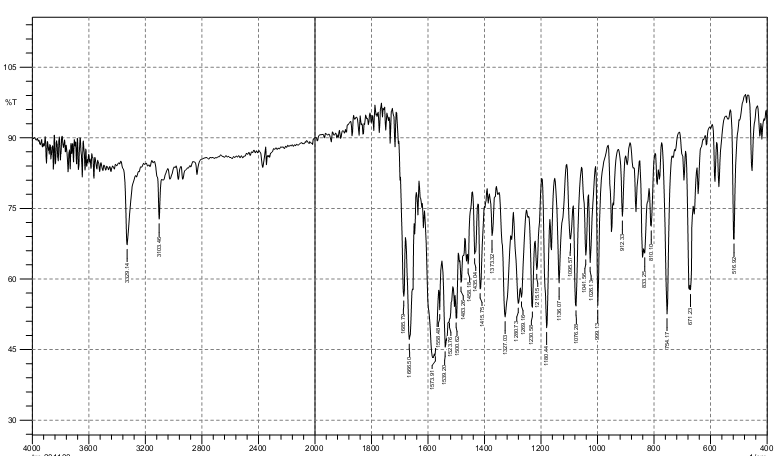

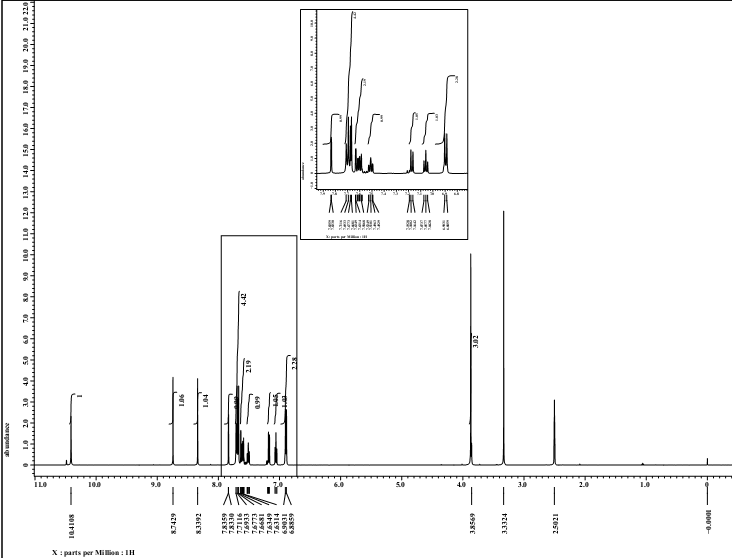

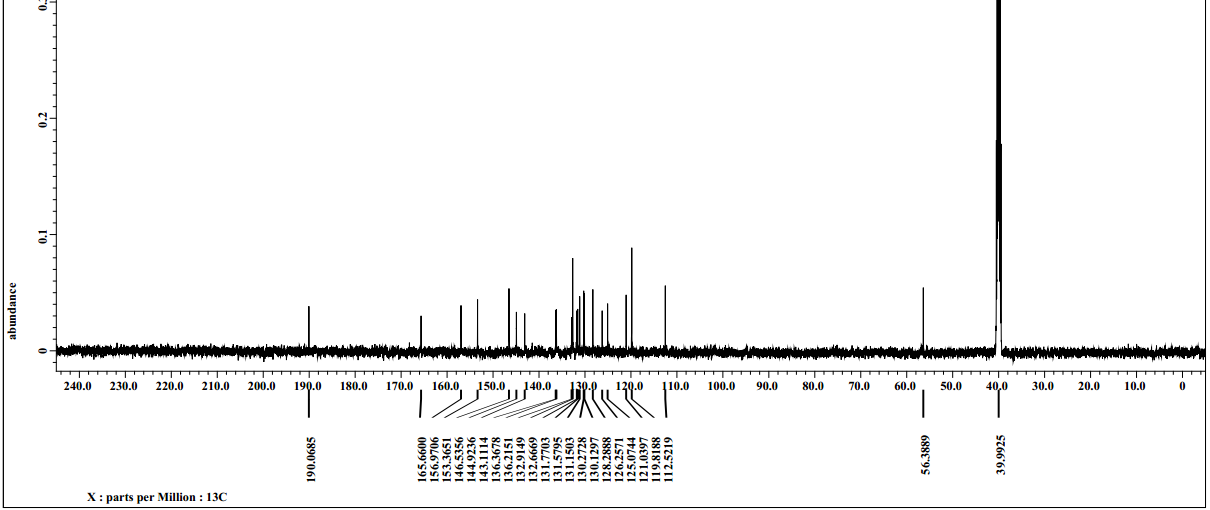

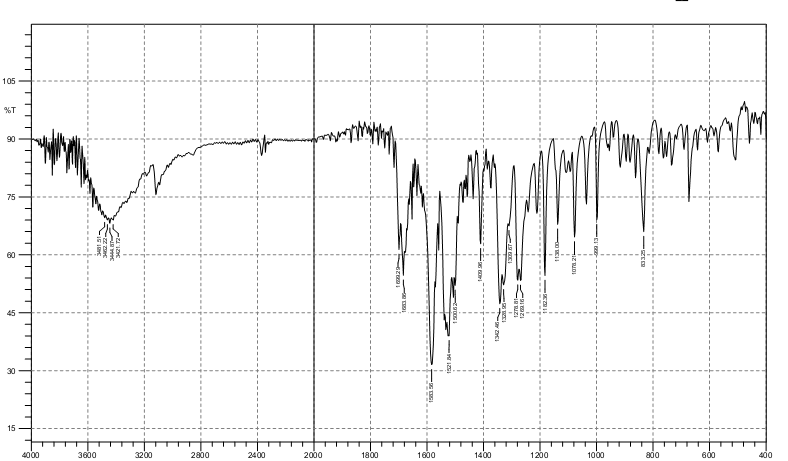

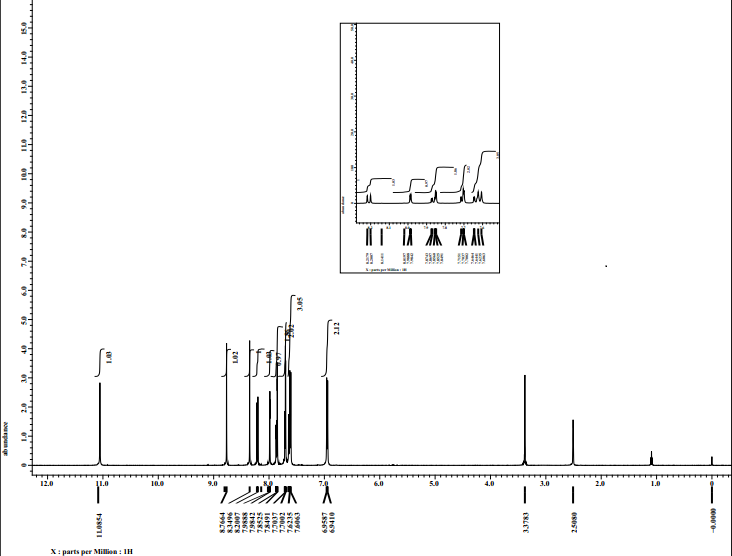

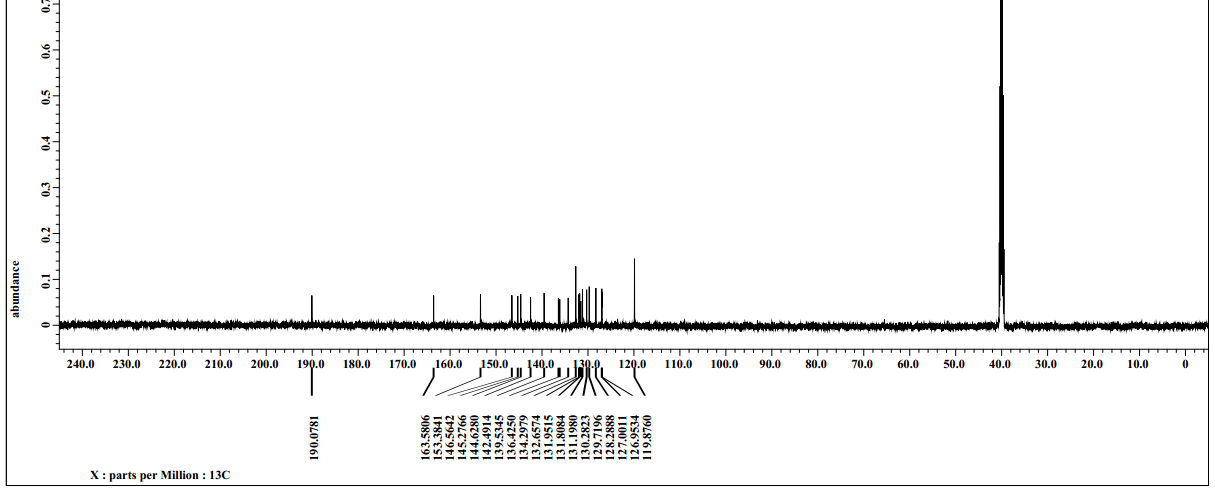

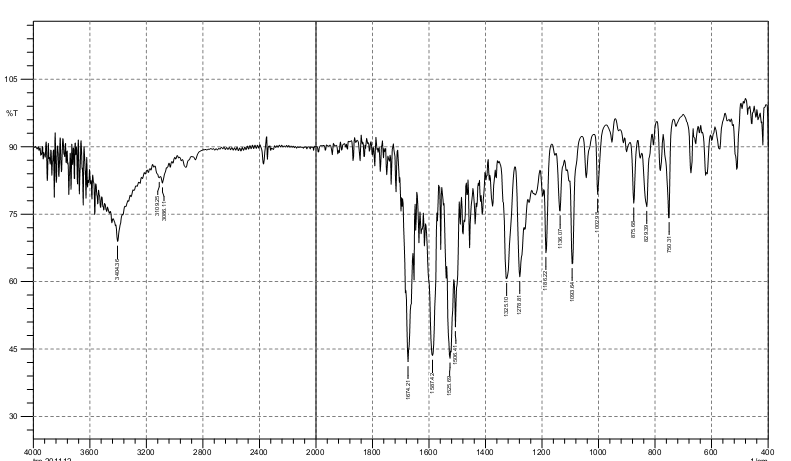

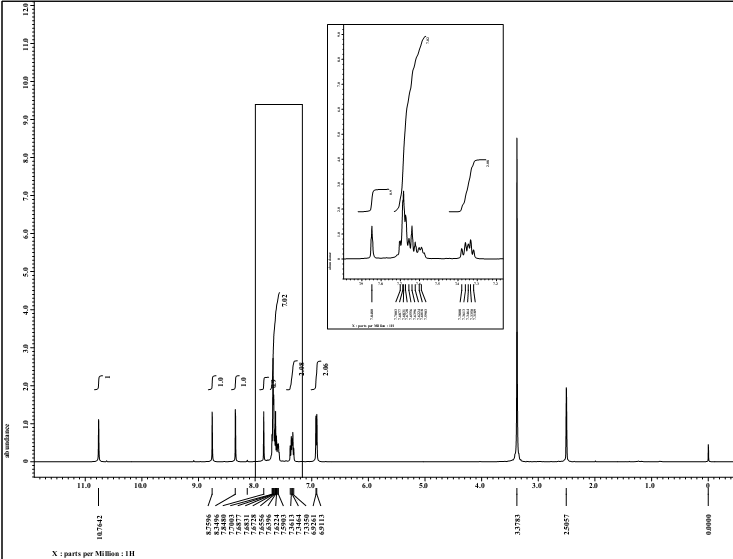

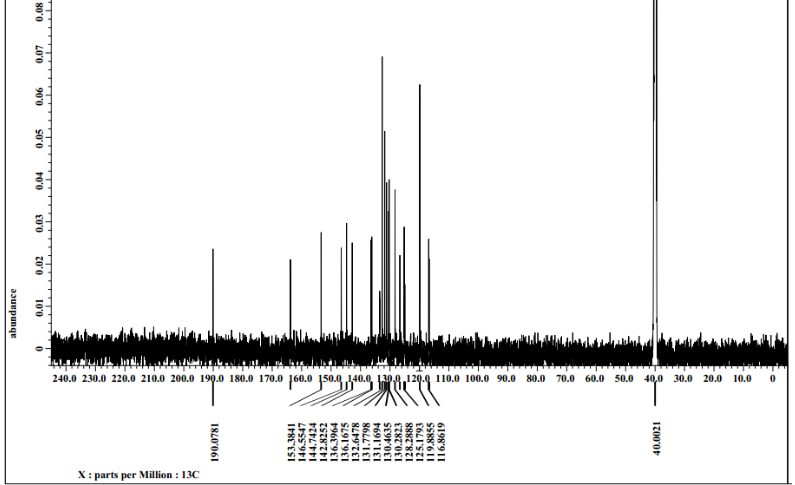

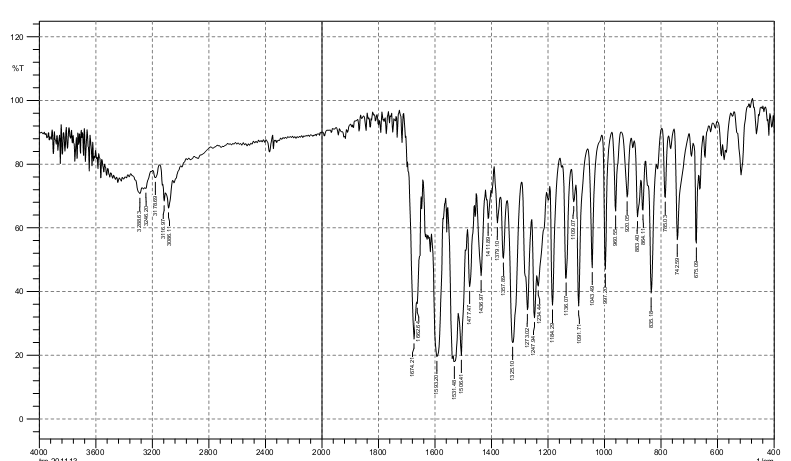

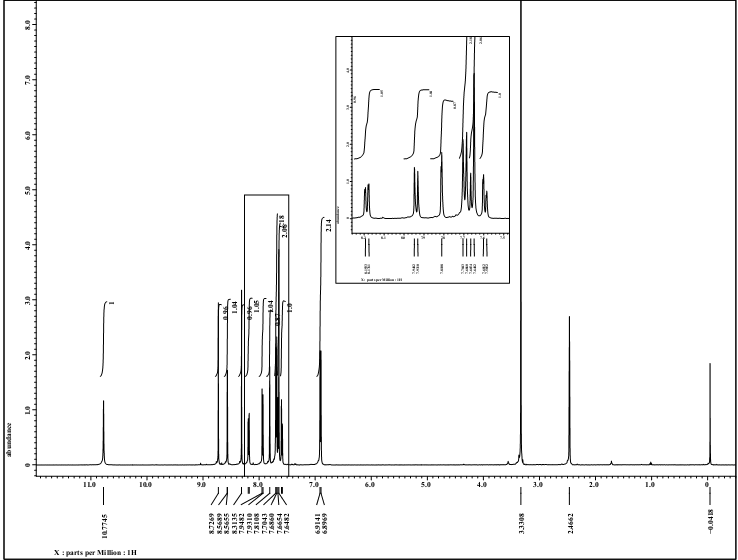

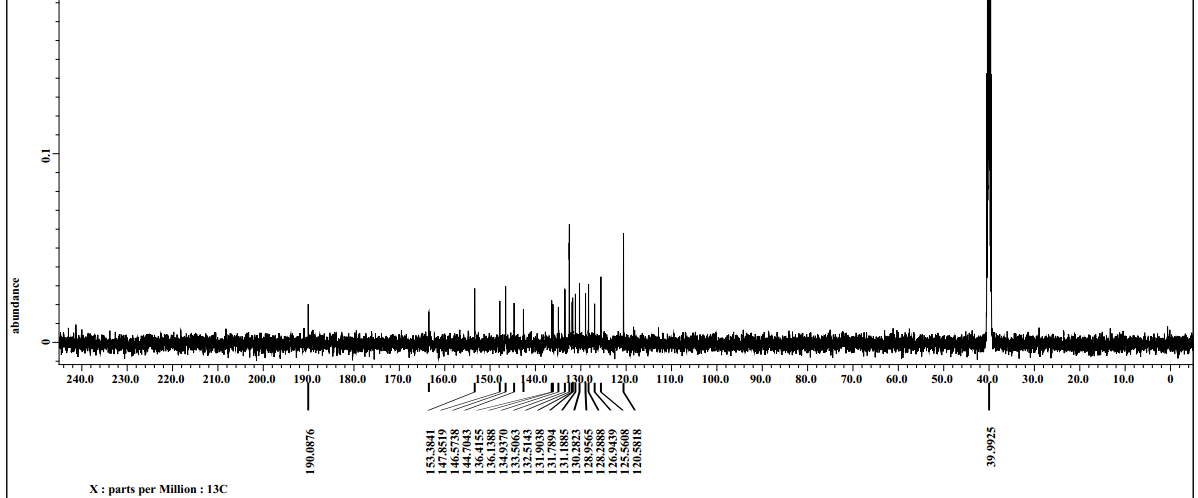

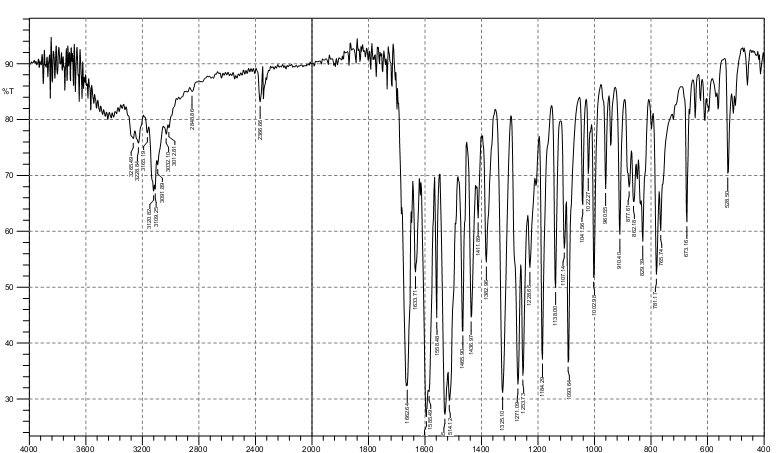

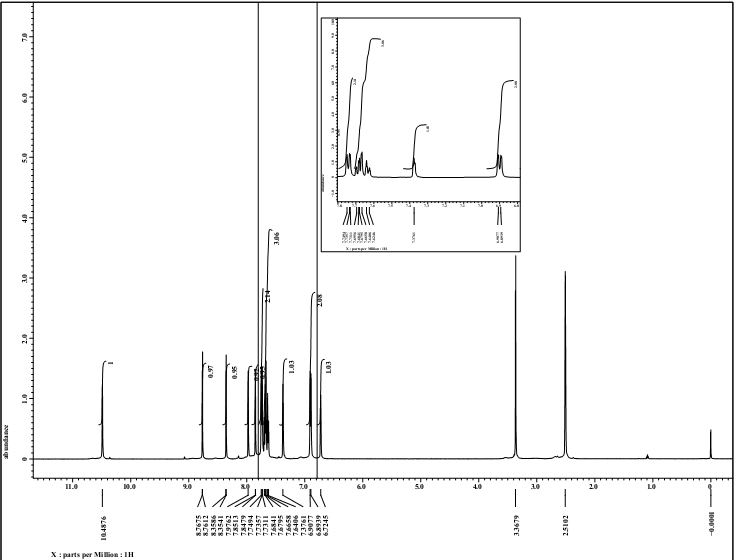

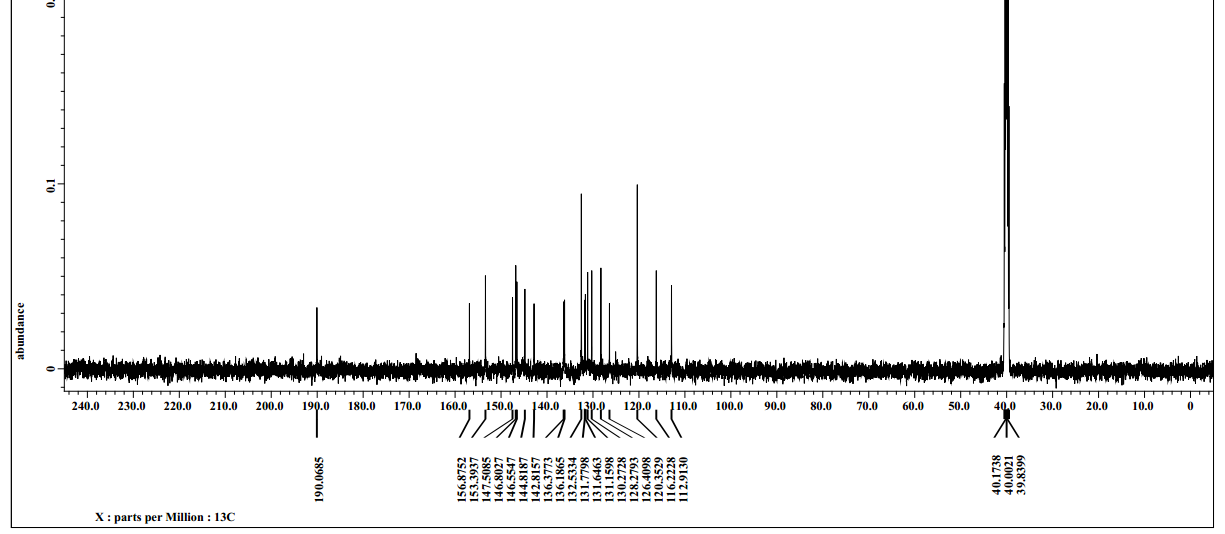

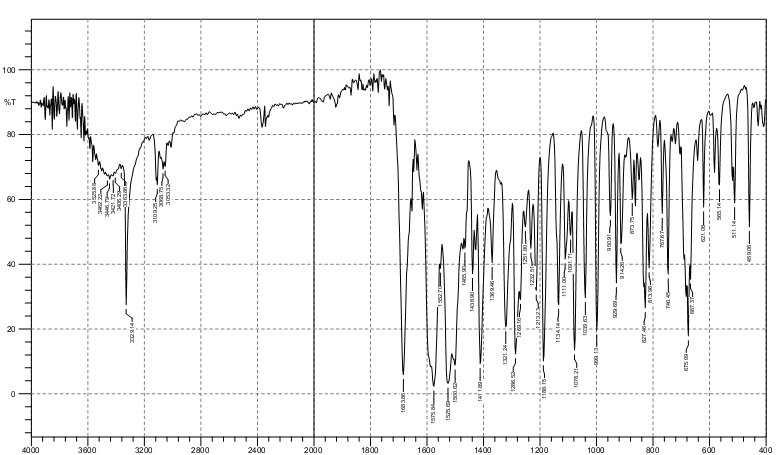

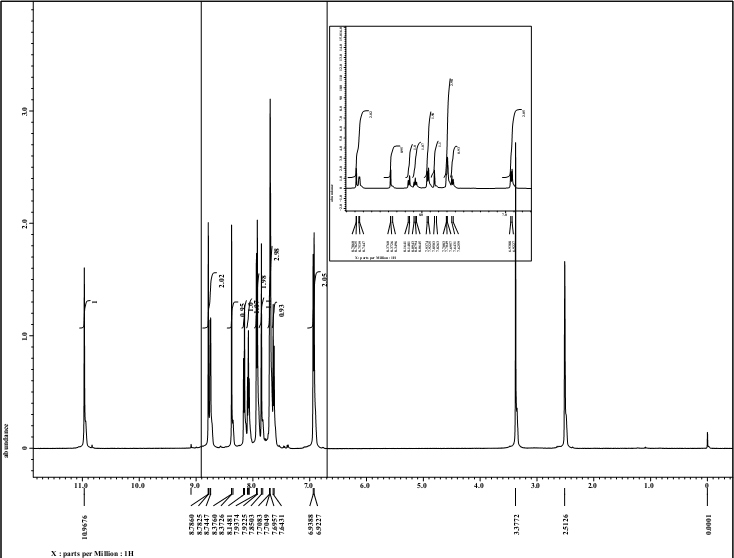

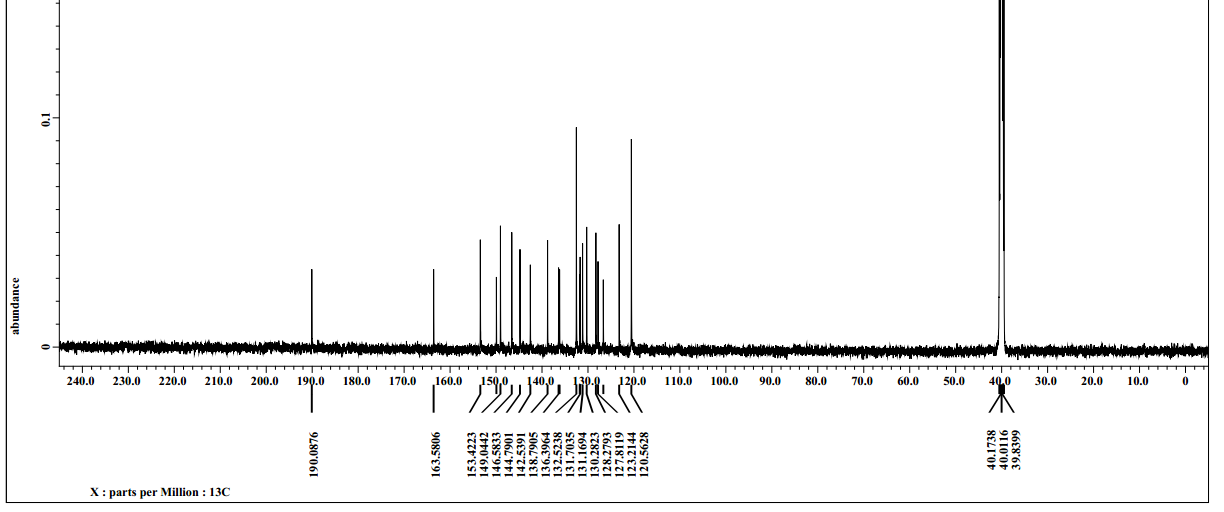

Supplement: Additional file 3 — Experimental details and data of the title compounds 4a to 4v. Experimental procedure, spectroscopic data of intermediate 3, title compounds 4a to 4v, copies of 1H NMR, 13C NMR, and IR spectroscopy. [file 1752-153X-7-30-S3.doc]
